# Proteomic Profiling of Pre- and Post-Surgery Saliva of Glioblastoma Patients: A Pilot Investigation

**DOI:** 10.3390/ijms252312984

**Published:** 2024-12-03

**Authors:** Alexandra Muntiu, Fabiana Moresi, Federica Vincenzoni, Diana Valeria Rossetti, Federica Iavarone, Irene Messana, Massimo Castagnola, Giuseppe La Rocca, Edoardo Mazzucchi, Alessandro Olivi, Andrea Urbani, Giovanni Sabatino, Claudia Desiderio

**Affiliations:** 1 Dipartimento di Scienze Biotecnologiche di Base, Cliniche Intensivologiche e Perioperatorie, Università Cattolica del Sacro Cuore, 00168 Rome, Italy; alexandra.muntiu@gmail.com (A.M.); federica.vincenzoni@unicatt.it (F.V.);; 2 Fondazione Policlinico Universitario A. Gemelli IRCCS, Catholic University, 00168 Rome, Italy; 3 Department of Neurosurgery, Mater Olbia Hospital, 07026 Olbia, Italy; edoardo.mazzucchi@gmail.com; 4 Istituto di Scienze e Tecnologie Chimiche “Giulio Natta”, Consiglio Nazionale delle Ricerche, 00168 Rome, Italy; dianaross79@hotmail.com (D.V.R.); imessana53@gmail.com (I.M.); 5 Centro Europeo di Ricerca sul Cervello-IRCCS Fondazione Santa Lucia, 00179 Rome, Italy; 6 Institute of Neurosurgery, Fondazione Policlinico Universitario A. Gemelli IRCCS, Catholic University, 00168 Rome, Italy

**Keywords:** bottom-up proteomics, glioblastoma multiforme, saliva, filter-aided sample preparation, brain tumor, proteomics, biomarkers

## Abstract

Glioblastoma multiforme (GBM) is an extremely aggressive brain tumor characterized by a high infiltration capability and recurrence rate. Early diagnosis is crucial to improve the prognosis and to personalize the therapeutic approach. This research explored, by LC-MS proteomic analysis after proteolytic digestion, the molecular profile of pre- and post-operative saliva pools from newly diagnosed (ND) GBM patients by comparing different times of collection and tumor recurrence (R). CYCS, PRDX2, RAB1C, PSMB1, KLK6, TMOD3, PAI2, PLBD1, CAST, and AHNAK, all involved in processes of tumor invasiveness and chemo- and radio-resistance, were found to depict the pre-surgery saliva of both ND and R GBM. PADI4 and CRYAB proteins, identified among the most abundant proteins exclusive of ND GBM pre-surgery saliva and classified as proteins elevated in glioma, could have a potential role as disease biomarkers. Selected panels of S100 proteins were found to potentially differentiate ND from R GBM patient saliva. TPD52 and IGKV3, exclusively identified in R GBM saliva, could be additionally distinctive of tumor relapse. Among the proteins identified in all pools, label-free relative quantitation showed statistically significant different levels of TXN, SERPINB5, FABP5, and S100A11 proteins between the pools. All of these proteins showed higher levels in both ND_ and R_T0 pre-surgery saliva with respect to CTRL and different modulation after surgery or chemo-radiotherapy combined treatment, suggesting a role as a potential panel of GBM predictive and prognostic biomarkers. These results highlight and confirm that saliva, a biofluid featured for an easily accessible and low invasiveness collection, is a promising source of GBM biomarkers, showing new potential opportunities for the development of targeted therapies and diagnostic tools.

## 1. Introduction

Glioblastoma IDH-wild type grade 4, also known as glioblastoma multiforme (GBM), is the most invasive brain cancer due to its high infiltration capability and relapse phenomena, which make this disease a challenge in research studies as well as in the clinic [1,2]. Indeed, despite advances in the fight against cancer, GBM represents a very aggressive malignant tumor of the brain with a low survival rate [1,2,3,4]. Progress in the knowledge of the GBM molecular profile is fundamental for the discovery of molecular targets for the development of therapies and biomarkers for clinical purposes in diagnostics [5]. GBM diagnosis is nowadays principally based on neuroimaging [3], and surgery is currently the only treatment modality, followed by co-adjuvant radio-chemotherapy, usually employing temozolomide [6]. The discovery of rapid and minimally invasive diagnostic tools is, therefore, in high demand.

From this perspective, this work aimed to perform the first proteomic investigation, to the best of our knowledge, on newly diagnosed (ND) GBM patients’ saliva pools collected at different times from diagnosis, i.e., before the surgical removal of the tumor (T0) and one (T1) and three months (T3) post-operatively. Alterations of the salivary protein profile over time and treatments could allow the identification of potential predictive and prognostic disease biomarkers to be further investigated and validated on a larger cohort of individual samples for clinical applications. Specifically, the T1 and T3 post-surgery saliva samples were collected during the patient follow-up before and after radio-chemotherapy treatment, respectively. The obtained results were then compared with sample pools of saliva from patients affected by GBM tumor relapse (R) collected before the tumor removal (T0 saliva) and with control (CTRL) samples.

Saliva is a biofluid that is easy to collect, with good patient compliance and high diagnostic potential [7]. Saliva is not a sterile fluid, and many factors can affect its composition, i.e., the method of collection (unstimulated—holding the saliva for a few seconds and splitting it into a vessel or stimulated—collected by mastication) [8], the patient age (children and adults present several differences in the protein profile) [9,10], the time of collection (in the morning, saliva is roughly composed in equal part by the secretions of parotid and submandibular/sublingual glands) [7], and storage [7,8], which need to be carefully considered. About 60% of the total human proteins are expressed in saliva [11]; 90% of them are secreted by the major salivary glands and 10% by the minor salivary glands or derived from the gingival crevicular fluid [12]. Saliva is a biological fluid advantageously less complex with respect to blood or serum [13], the latter characterized by highly abundant proteins interfering with and making challenging the characterization of low-abundance small proteins and peptides, which are a source of potential biomarkers and molecular targets of new therapeutics [14]. As with serum and blood, the concentration of the salivary proteins can change in the presence of systemic diseases, making this biofluid a potential source of biomarkers and an ideal biological matrix candidate for diagnostic applications to be investigated. Indeed, saliva has already been reported to be a source of cancer biomarkers [15,16,17]. Particularly, Haptoglobin, Zinc-2-glycoprotein, and Calprotectin were reported as salivary biomarkers of lung cancer [15]. Cystatin B, Triosephosphate isomerase, and Malignant brain tumors 1 protein were described as salivary biomarkers of gastric cancer [16]. Salivary Cancer antigen, Erythroblastic oncogene B, and Vascular endothelial growth factor were instead reported as breast cancer biomarkers [17].

The results of the present pilot investigation revealed distinct proteomic profiles of the acid-soluble fraction of saliva samples from ND and R GBM patients that were analyzed after protein digestion. Due to the difficulty of collecting saliva at the same times from the same patients in the post-surgery follow-up, the study involved a limited number of specimens that were analyzed as pools for a pilot overview of the pre- and post-surgery proteomic profile of ND and R GBM patient saliva through the evaluation of label-free quantitative data, grouping analysis, gene ontology classification, and functional network analysis.

## 2. Results and Discussion

The acid-soluble fractions of saliva samples collected at different times from GBM patients were pooled and preliminarily explored by LC-MS proteomic analysis after FASP digestion to investigate potential differences in the molecular profiles associated with the tumor type, i.e., ND versus R GBM, and time of collection, i.e., pre- and post-surgery, for potential diagnostic applications in precision medicine. The analysis of sample pools minimized the inter-individual variability to allow an initial proteomic study. Pools were prepared by combining the same total protein quantity of the single specimens grouped by type in order to allow qualitative and quantitative data comparisons using a label-free approach by processing and analyzing the same protein content for each pool and averaging the data from the three analytical replicates. Statistically significant differences in label-free protein area values obtained from software processing of raw LC-MS data were assessed by *t*-test or one-way ANOVA with Tukey’s post hoc test for multiple sample comparison, as specified each time, and considering a *p*-value less than or equal to 0.05 as significant.

In detail, saliva from three ND GBM patients was collected before the tumor removal (T0 saliva) and during the follow-up at one (T1 saliva) and three (T3 saliva) months post-surgery. Specifically, T3 saliva was collected after radio-chemotherapy combined treatment. Saliva was also collected from three patients affected by GBM tumor relapse (R) before surgery (R_T0) and pooled to be analyzed in comparison with ND_T0 saliva. All data were finally compared with data from a pool of saliva from healthy controls (CTRL).

Lists of the proteins identified in the saliva pools were obtained from multireport data elaboration of LC-MS analytical triplicates after the application of stringent filters to the results following the Human Proteome Project (HPP) mass spectrometry data interpretation guidelines [18] (see Section 3 for details). The data are reported in Table S1A–E. These filters ensured high-confidence identification data, although the number of identifications was considerably reduced compared to the total number of identifications obtained.

**Figure 1:**
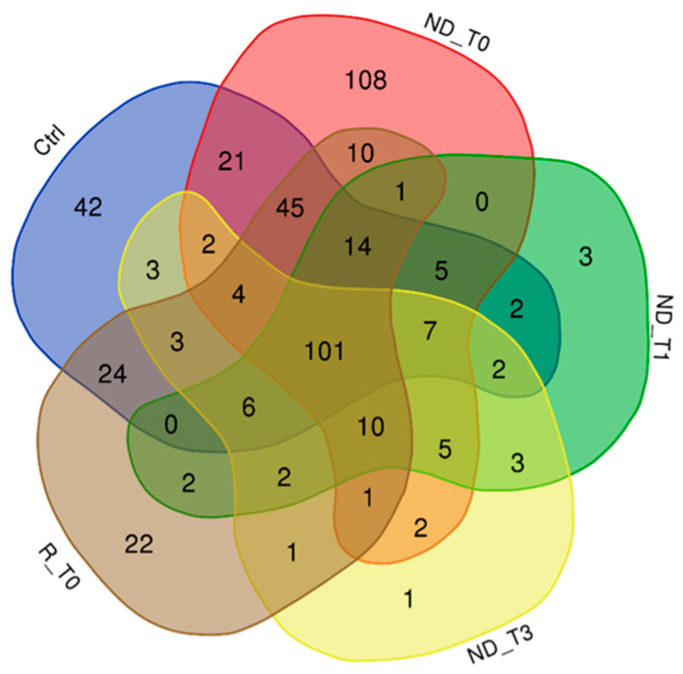
Venn diagram resulting from grouping analysis of the proteins identified in ND_T0, T1, and T3 GBM saliva versus R_T0 GBM and CTRL saliva.

Figure 1 reports the Venn diagram resulting from grouping analysis of the proteins identified in each pool, namely, 336 proteins for ND_T0 saliva (Table S1A), 163 for ND_T1 (Table S1B), 153 for ND_T3 (Table S1C), 246 for R_T0 (Table S1D), and 281 for CTRL (Table S1E). Overall, 452 proteins were identified, which were differently distributed across pools, evidencing exclusive and shared proteins in each condition. These proteins, described and compared in detail in the following paragraphs, were analyzed by bioinformatic tools.

### 2.1. Proteins Exclusive of ND_ and R_T0 Saliva

In the attempt to identify the proteomic profile of GBM tumor saliva, attention was first directed to the proteins identified in the T0 pre-surgery saliva pools of ND and R GBM. As shown in Figure 1, these pools shared the identification of 10 proteins, listed in Table 1, with molecular mass ranging from 11 to 628 kDa. These proteins, since identified exclusively in both ND_ and R_T0 pools, can be considered to depict the core molecular profile of GBM tumor saliva. Among them, it is noteworthy to underline that four proteins (bold in Table 1), namely, peroxiredoxin-2 (PRDX2), kallikrein-6 (KLK6), plasminogen activator inhibitor 2 (PAI2), and calpastatin (CAST), are classified as Cancer Related genes in the Human Protein Atlas database [19,20,21] (https://www.proteinatlas.org/search/protein_class:Cancer-related+genes, 5 August 2024) and in addition, with the exception of CAST, as Candidate Cancer Biomarkers (https://www.proteinatlas.org/search/protein_class:Candidate+cancer+biomarkers, accessed on 5 August 2024). Interestingly, in our previous investigation [22], some of these proteins have been identified in GBM Cavitron Ultrasonic Surgical Aspirator (CUSA) fluid collected from different zones of ND and R tumors, i.e., tumor CORE and tumor periphery either positive (A+) or negative (A−) to 5-aminolevulinic acid-induced fluorescence, as reported in Table 1.

**Figure 2:**
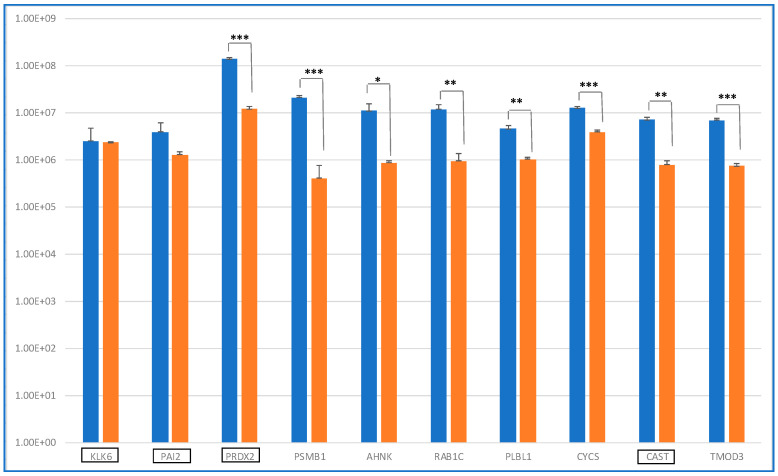
Bar chart (log scale) of the label-free relative quantitation of protein area values (mean value of replicate LC-MS analysis ± SD) in ND (blue) and R (orange) T0 saliva of the 10 shared proteins identified (Table 1). On the *x*-axis, the proteins classified as Cancer Related are highlighted by a black rectangle. Significant differences have been determined by *t*-test (***, *p* value < 0.01; **, *p* value < 0.05; *, *p* value < 0.1).

It was at first interesting to compare the levels of these 10 distinctive proteins in ND_ and R_T0 saliva by label-free relative quantitation of the protein area values calculated by the Proteome Discoverer (version 1.4.1.14) software elaboration of the MS raw data. Figure 2 shows the bar chart (in log scale) of the protein areas, as average value ± SD of analytical triplicates, with the results of the *t*-test of the statistically significant differences.

Eight proteins showed levels with statistically significant differences between ND_ and R_T0 saliva, with four of them exhibiting a highly significant alteration (*** *p*-value < 0.01), namely, PRDX2, PSMB1, CYCS, and TMOD3. All four of these proteins showed higher levels in ND_T0 saliva with respect to R_T0 saliva. The difference between ND_ and R_T0 saliva was particularly high for PRDX2.

KLK6 and PAI2, both classified as Cancer Related proteins, were the unique proteins of this group with similar levels in ND_ and R_T0 saliva. Kallikrein-6 (KLK6) belongs to a subgroup of serine proteases, and it has a promising role as a diagnostic or prognostic biomarker, given its involvement in cancer progression [23]. KLK6 demonstrated a diagnostic potential for laryngeal cancer, as it was a candidate biomarker in plasma samples [24]. KLK6 was reported to directly promote the survival of glioma cells, contributing to their resistance to both radiation and temozolomide treatments through a PAR1-dependent mechanism [25]. The identification of KLK6 in both ND_ and R_T0 saliva, with similar area values, is particularly interesting because this protein is classified as highly expressed in the brain according to The Human Protein Atlas. As reported by Ghosh et al., KLK6 could play a role in tumor cell invasion and metastasis and could be a therapeutic target candidate [26].

Plasminogen activator inhibitor 2 (PAI2 or SERPINB2) is linked to the tumorigenesis of several cancers. Higher levels of the protein have been reported to correlate with a higher rate of tumor metastasis/recurrence and lower overall survival [27]. Overexpression of PAI2 has been found in cancer stem cells, the cells responsible for tumor drug resistance and recurrence, making this protein an interesting molecular target to be further investigated in GBM. U138 glioblastoma cells were found to produce PAI-2 [28]. Single-Nucleotide Polymorphisms (SNPs) in the Serpin B family proteins have been studied in correlation with the prognosis of GBM patients; however, this study did not specifically investigate SERPINB2 [29].

The most abundant Cancer Related protein found in ND_T0 saliva was PRDX2, a protein that protects cells from oxidative stress, which is a hallmark process of cancer [30]. PRDXs have a regulatory role in the signaling pathways related to cancer development. Particularly, PRDX2 was found to be overexpressed in several cancers and associated with cancer progression and poor overall survival. Reactive oxygen species (ROS) generation is involved in tumor progression and therapy resistance of GBM [31] and, particularly, the level of expression of PRDX2 was correlated with tumor resistance to radio- or chemotherapy treatments [32]. Müller Bark et al. [33] demonstrated the presence of PRDX2 in extracellular vesicles (EVs) of GBM saliva. Indeed, diverse studies have demonstrated the presence of EVs in the plasma and saliva of glioma patients [33,34], an interesting finding considering the ability of EVs to cross the blood–brain barrier and transport glioma biomarkers [35].

Calpastatin (CAST), the fourth Cancer Related protein of Table 1, is a specific inhibitor of calpain, a protein that suppresses GBM tumor growth and invasion through the cleavage of filamin A [36]. The calpain/calpastatin proteolytic system has been studied in relation to GBM [37]. A phospho-proteomic study compared the response of GBM cell lines to radiation with respect to normal human astrocytes, and CAST phosphorylation was found to be involved in GBM cell radiation resistance through the promotion of cell survival and invasion by modulating calpain protease activity [38]. The detection of CAST in the saliva of patients affected by GBM is noteworthy, given the role outlined for this protein in GBM. This finding highlights the importance of CAST in the context of tumor suppression mechanisms and may provide new insights into GBM pathology.

According to the Human Protein Atlas database (accessed on 6 August 2024) and to the biological matrix analyzed, the 10 proteins exclusive of ND_ and R_T0 saliva pools are all included, with the exception of putative Ras-related protein Rab-1C (RAB1C) in the list of the proteins “expressed in salivary gland”, a class accounting for a total of 14,348 transcripted genes. RAB1C, a small protein of about 22 kDa in saliva, seems, therefore, to not originate from salivary gland secretion. This membrane protein, with alternative names of RAY and RAB35, showed increased levels in inflammatory breast cancer cells with respect to normal cells [39]. Furthermore, RAB1C is an oncogenic Rab GTPase, a subfamily of the Ras proteins with a key role in membrane trafficking, actin dynamics, and the regulation of vesicular trafficking, processes that all play a relevant role in cancer [40,41]. A recent study reviewed the biological functions of RAB35 and its role in cancer invasion, metastasis, and immune evasion, with a special focus on leukemia [42]. The role of RAB35 in modulating actin dynamics, vesicular trafficking, GTPase interactions, exosome release, and the PI3K/AKT pathway could be the basis of the protein’s involvement in the development and invasion of leukemia. Alteration of RAB35 could affect cancer through different mechanisms, such as the release of tumor cells through vesicle trafficking and the regulation of cell adhesion and survival through recycling beta1-integrin, cadherins, and EGF receptor membrane proteins. In GBM, RAB35 seems downregulated [43]. Indeed, in mice implanted with GBM cancer stem cells, RAB35 down-expression produced tumor growth, while its overexpression decreased tumor development and spread [43]. An ARF5/RAB35 signaling cascade has been shown to regulate GBM growth and invasiveness, and the knockdown of both RAB35 and ARF5 increased the distance and velocity of glioma cell migration in in vitro studies. The loss of function of either ARF5 or RAB35 produced enhanced recycling and activation of EGFR and then of SPOCD1, generating cancer phenotypes. The upregulation of SPOCD1 drove the proliferation and metastasis of glioma cells, in part via upregulation of Pentraxin 3 [43].

A very recent paper reviewed the role of neuroblast differentiation-associated protein (ANHAK) in physiological states and malignant tumors, including glioblastoma [44]. ANHAK is a large protein, approximately 700 kDa, that plays a role in various biological processes. These include the regulation of calcium channels, the formation of the blood–brain barrier, muscle membrane repair, embryonic development, fat metabolism, and inflammatory responses. Its peptide fragment 5758–5775 was reported to activate the release of IL-8 and TNF-α from mast cells. ANHAK is down-expressed in glioma cell lines with respect to normal glial cells. Acting as a tumor suppressor, low levels of the proteins were correlated to poor prognosis in GBM, while in other tumors, the proteins showed, in contrast, overexpression. The role of ANHAK in GBM is still an interesting topic of investigation, with the presence of the mutated proteins apparently correlated to an even worse prognosis. It is interesting that the AHNAK2 gene (of the ANHAK family) has recently been found to be among the top ten altered genes commonly found in CGGA, TCGA, CPTAC, and MAYO-PDX glioblastoma datasets [45]. Mutation of AHNAK2, in combination or not with mutation of the OBSCN gene, was associated with a better overall survival.

Cytochrome C (CYCS), a protein involved in the maintenance of cell life through respiration and in the apoptotic cascade, was found to be down-expressed in glioma tissues with respect to normal samples: the lower the expression, the higher the glioma grade. As a result, CYCS was evidenced as a potential prognostic biomarker of glioma since its low expression seems to be correlated with tumor progression [46]. Very recent papers published in the current year investigated the role of cytochrome C and mitochondrial metabolism in GBM [47,48].

Proteasome subunit beta type-1 (PSMB1) is one of the seven proteins that are members of the 20S proteasome PSMB1–7. PSMB1 was reported among the top upregulated genes in bladder cancer tissue with respect to normal tissue [49]. PSMB 1/2/3/4/6/8/9/10 have been reported as biomarkers of prognosis in Clear Cell Renal Cell Carcinoma [50]. The role of proteasomes in glioma cell survival has long been studied [51]. Different studies remark on the role of different PSMB members in GBM, with PSMB2 expression reported to correlate with high-grade glioma and poor prognosis [52] and PSMB4 decreased expression with the increased therapeutic effects of temozolomide [53]. Specific studies are instead needed to investigate the role of PSMB1 more deeply.

The clinical prognostic role of Phospholipase B-like 1 (PLBD1) was investigated in different cancers by studying data of expression, methylation, and mutations, and a special focus was dedicated to glioma [54]. The overexpression of PLBD1 was recognized in several cancers. Particularly in relation to glioma, PLBD1 was found to affect the immunological profile and to promote tumor invasiveness and proliferation, making the protein a potential prognosis biomarker and immunotherapeutic target.

Tropomodulin isoforms are cytoskeleton regulatory proteins that cover the end filaments of F-actin in a tropomyosin-dependent manner [55]. Tropomodulin isoforms sequester specific monomeric isoforms of F-actin, resulting in different localization and mechanisms of actin remodeling, which is a known molecular mechanism implicated in cancer [56]. Tropomodulin-3 (TMOD3), particularly, is mainly present in contexts of cellular dynamics, such as migrating endothelial cells or proliferating erythroblasts. Recently, TMOD3 was identified as a substrate of asparagine endopeptidase—an enzyme playing a role in cancer with its catalytic activity and producing different truncated forms of TMOD3—and was detected in diverse tumors and associated with poor prognosis, particularly in high-grade glioma. The asparagine endopeptidase—TMOD3 protease signaling axis was, therefore, found to be involved in the regulation of tumor cell proliferation and migration, resulting in an interesting target for molecular therapies [57].

It is noteworthy to mention that not one of the proteins in Table 1 is classified as a product of “genes associated with unfavorable and favorable prognosis” in GBM, “genes with elevated expression in glioma” with respect to other cancers, and “genes only detected in glioma” (https://www.proteinatlas.org/humanproteome/cancer/glioblastoma+multiforme, accessed on 6 August 2024). It is interesting to underline that PRDX2 was identified in the CUSA fluid collected from all tumor zones, including the CORE and periphery, of both ND and R GBM [22]; thus, the protein seems to mark all the GBM biological specimens analyzed. The other three proteins out of the ten in Table 1 have been previously characterized in GBM CUSA fluid, namely CYCS, identified exclusively in the ND and R A^+^ tumor peripheral zones; RAB1C, identified in the ND A+ tumor periphery; and CAST, interestingly identified in the ND non-tumoral peripheral tissue (A− tumor periphery) and in the A+ tumor periphery of recurrence. These proteins are, therefore, suggested to be further investigated as GBM diagnostic biomarkers in saliva, marking the tumor zone. Particularly, CAST, present in the A− periphery of the ND tumor, could be involved in the onset of tumor relapse, where the protein characterizes, in contrast, the A+ tumor zone. While the proteins identified in both ND_ and R_T0 saliva can be considered to represent the pathological molecular profile of GBM saliva, the proteins exclusively depicting the ND_ or the R_T0 saliva could, on the other hand, disclose potential GBM biomarkers of diagnosis or early recurrence, and they could provide a deeper understanding of the molecular mechanisms of disease progression and relapse. These proteins will be described and discussed below in separate paragraphs.

### 2.2. Proteins Exclusive of ND_T0 Saliva

As shown in Figure 1, 108 proteins of molecular mass ranging between 7.8 and 226.4 kDa were exclusively identified in ND_T0 saliva (Table S2). It is noteworthy that 52 of these proteins (marked with “*”) were identified in GBM CUSA fluid in our previous investigation [22], and 24 are classified as Cancer Related (bold in Table S2).

**Figure 3:**
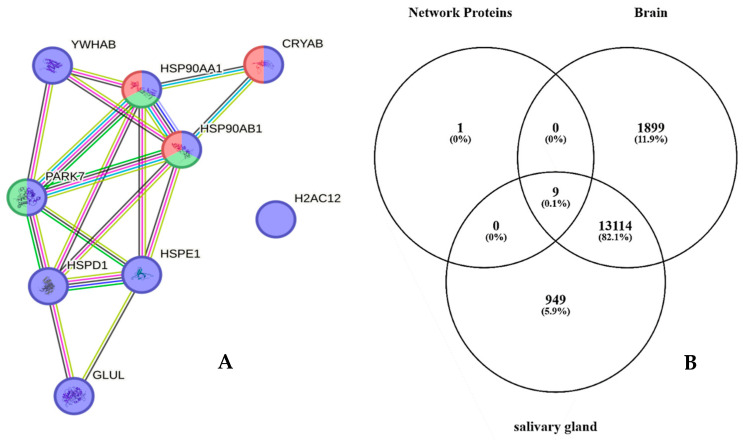
(**A**) STRING tool protein functional network analysis of HSPE1, H2AC12, PARK7, CRYAB, YWHAB, LDHB, GLUL, HSPD1, HSP90AA1, and HSP90AB1, exclusive of ND_T0 saliva and also identified in GBM CUSA fluid from all tumor zones in our previous paper [19]. Node colors specify proteins in extracellular exosomes (purple nodes), involvement in Chaperone-Mediated Autophagy (green nodes), and HSF1-dependent transactivation (red nodes). The edges represent protein-protein associations, with each color indicating a specific type: known interactions (light blue: curated databases, purple: experimentally determined); predicted interactions (green: gene neighborhood, red: gene fusions, blue: gene co-occurrence); others (ochre: textmining, black: co-expression, light blue: protein homology). (**B**) Venn diagram resulting from grouping analysis of these ten proteins versus the list of brain and salivary gland classified proteins in the Human Protein Atlas database.

Particularly, ten proteins out of this group, namely, HSPE1, H2AC12, PARK7, CRYAB, YWHAB, LDHB, GLUL, HSPD1, HSP90AA1, and HSP90AB1, have been characterized in GBM CUSA fluid from all tumor zones, either CORE or periphery, of both ND and R_GBM [22]. These proteins, therefore, depict ND_T0 saliva as well as the tumor tissue, including the A− zone, the latter finding suggesting their potential involvement in tumor progression and recurrence. Interestingly, analysis of their functional interactions by the STRING tool revealed that nine out of these ten proteins are highly interconnected and that specific cellular components and pathways were enriched in the network, namely, extracellular exosomes (purple nodes) (False Discovery Rate, FDR, value 3.65 × 10^−6^), Chaperone-Mediated Autophagy (green nodes) (FDR value 0.00034), and HSF1-dependent transactivation (red nodes) (FDR value 0.00034) (Figure 3A). According to the Human Protein Atlas database, these proteins, apart from Histone H2A type 1-H (H2AC12), which is the unique disconnected node of the network, are all expressed both in the brain and salivary glands (Figure 3B), indicating their involvement in the functions of these tissues.

It is also interesting to underline that 6 proteins out of the 108, namely, IGHV3-9, H2AC12, HBG1, NACA, KLK14, and PADI4, are not classified as salivary gland proteins in The Human Protein Atlas Database; therefore, their presence in saliva could be linked to tumor pathology.

In the list in Table S2, special attention was furthermore devoted to alpha-crystallin B chain protein (CRYAB) due to its classification in The Human Protein Atlas database as a gene highly expressed in glioma. The co-identification of the protein in GBM tumor saliva and CUSA fluid [22] led us to hypothesize its potential role as a GBM biomarker, considering that it is also classified as both Cancer Related and a Candidate Cancer Biomarker in The Human Protein Atlas database. Previous studies documented the increased expression of CRYAB protein in GBM [58,59]; however, to the best of our knowledge, its identification in GBM saliva has never been reported to date. It is noteworthy that the CRYAB protein was among the most abundant proteins identified in GBM ND_T0 saliva (Table S1). Nowadays, it is recognized that high expression of CRYAB in GBM promotes tumor resistance to treatment as well as tumor survival and proliferation by aiding cancer cells in adapting to adverse environmental conditions and in alleviating cellular stress [60]. While the specific functions and interaction mechanisms of CRYAB in GBM remain only partially understood, the protein is regarded as a significant factor in the occurrence and progression of GBM, and its expression is associated with poor prognosis [60]. CRYAB could, therefore, represent a potential diagnostic/prognostic marker in tumor tissue [60,61], which, based on our findings, could be advantageously determined in low-invasiveness biofluids such as saliva.

Another interesting finding among the 108 proteins exclusively identified in ND_T0 GBM saliva was the identification of protein-arginine deiminase type-4 (PADI4), a protein overexpressed in various tumors, including gliomas [62,63]. As recently reported [64], protein citrullination mediated by PADI4 plays a role in tumor progression and metastasis, suggesting a potential role of a prognostic biomarker and/or therapeutic target for this protein. PADI4 significantly contributes to enhancing the growth of primary tumor cells and the formation of distant metastases, suggesting the potential treatment of tumors with its inhibitors [65].

ND_T0 saliva interestingly showed the exclusive identification of S100A10 and S100A14. Unlike other proteins of the S100 family, S100A10 is peculiar in that it does not bind Ca^2+^ ions. A very recent paper reviewed the biological roles of this unique S100 family member, highlighting its involvement in several hallmark processes of cancer, including cancer stemness, tumor chemoresistance, and metastasis [66]. High expression of S100A10 was found in high-grade glioma and associated with a worse prognosis. Particularly, the protein was found to have a role in immunosuppression and inflammatory responses, resulting in being a potential target for diagnostic purposes and new treatments [67,68]. S100A14 was interestingly included in the list of proteins exclusive of the endosome-derived exosome fraction of the conditioned medium of GBM cells, carrying cargos of proteins involved in the processes of cell–matrix adhesion, cell migration and aggressiveness, and resistance to chemotherapeutic treatments [69]. Following a recent study on colon-rectal cancer, S100A14 is suggested to be investigated as a predictive biomarker for anti-PD-1/PDL1 anticancer therapy, an interesting correlation considering the role in GBM of the PD-1/PD-L1 axis [70,71,72,73].

**Figure 4:**
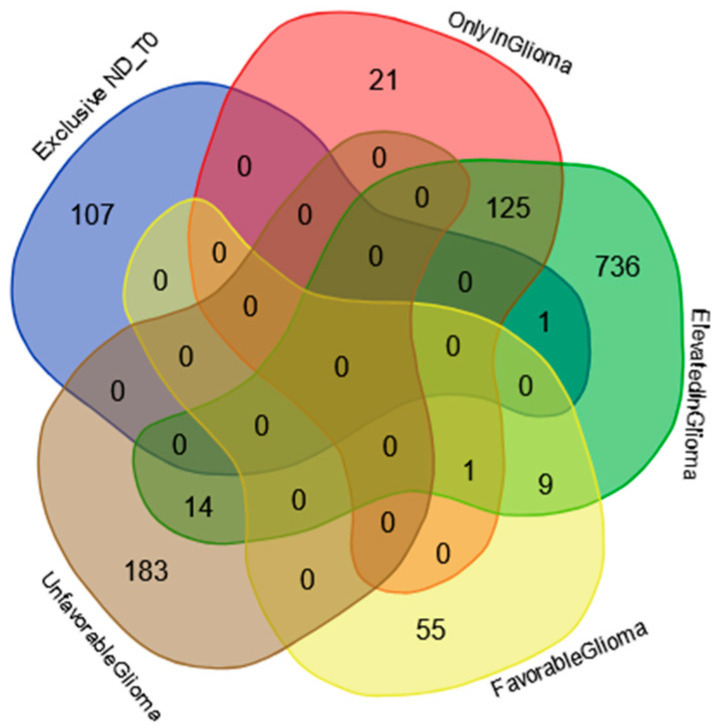
Venn diagram resulting from grouping analysis of the 108 exclusive proteins identified in the ND_T0 saliva pool versus different gene categories, namely, “gene elevated expression in glioma”, “gene only detected in glioma”, “unfavorable prognostic genes in GBM”, and “favorable prognostic genes in GBM”, downloaded from the Human Protein Atlas database.

The Venn diagram in Figure 4 shows the distribution of the 108 proteins exclusively identified in ND_T0 saliva (Table S2 list) among the specific gene classifications of The Human Protein Atlas database for glioma pathology (https://www.proteinatlas.org/humanproteome/cancer/glioblastoma+multiforme, accessed on 7 August 2024). Among the 108 proteins in the list, only CRYAB protein is the expression product of a gene with elevated expression in glioma, again highlighting the potential role of this protein in saliva as a GBM diagnostic biomarker, which should be deeply investigated.

**Figure 5:**
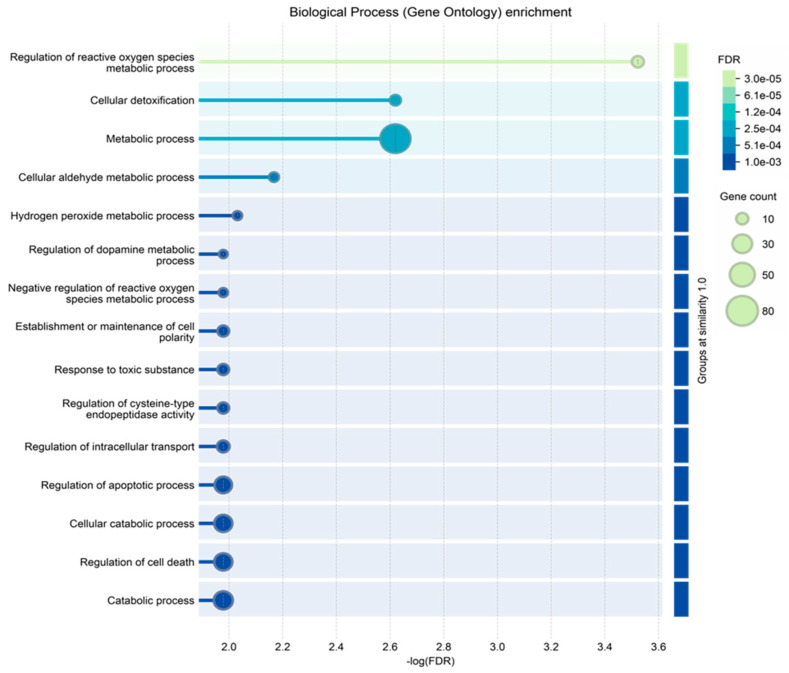
Biological process gene ontology enrichment analysis of the proteins characterized in ND_T0 saliva, including the 108 exclusive proteins and the 10 also identified in R-T0 saliva (top 15 results list).

**Figure 6:**
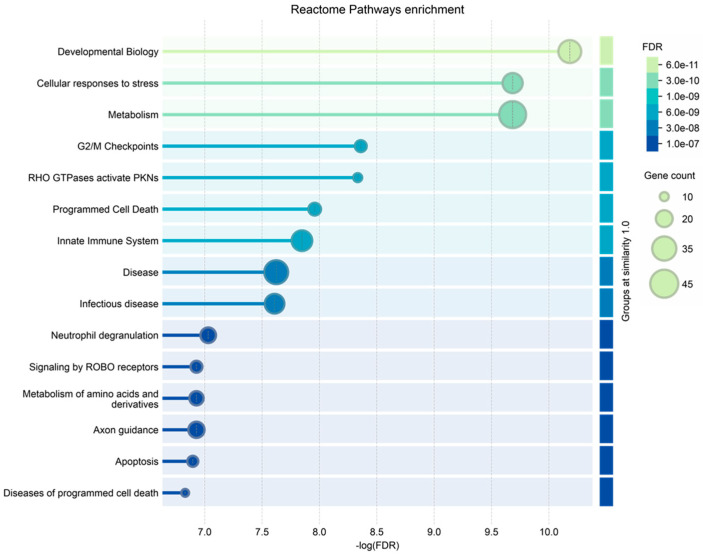
Reactome pathways enrichment analysis of the proteins characterized in ND_T0 saliva, including the 108 exclusive proteins and the 10 also identified in R-T0 saliva (top 15 results list).

Finally, the ND_tumor proteins, i.e., the 108 exclusive proteins (Table S2) and the 10 in common with R-T0 saliva (Table 1), were investigated by gene ontology enrichment analysis for biological process and reactome pathways by the STRING tool (Figure 5 and Figure 6, respectively). As depicted in Figure 6, the list of the top 15 most significantly enriched biological processes principally includes metabolic processes, in accordance with the important role of metabolism in the proliferation and growth of cancer cells, as well as in their survival and resistance to therapies; this has recently been reviewed for GBM, for which specific targeted therapies are under investigation [74,75,76]. This is also confirmed by the enrichment analysis of the molecular pathways, where metabolism is the third of the top 15 list (Figure 6), together with other pathways already reported as involved in brain tumor radio-resistance, such as the G2/M checkpoints pathway [77,78].

### 2.3. Proteins Exclusive of R_T0 Saliva

The proteins exclusively identified in R_T0 saliva represent a source of potential biomarkers for the early diagnosis of tumor recurrence during the post-surgery follow-up. This issue is very important for GBM, where diagnostic imaging must differentiate radiation necrosis from tumor recurrence. The identification of diagnostic biomarkers of early recurrence in a low-invasiveness biofluid, such as saliva, could significantly improve the patient prognosis and the clinical approach in support of diagnostic imaging.

A well-known concept in oncology is that tumor relapse often presents a molecular profile distinct from the primary newly diagnosed tumor [79,80]. In fact, the proteomic analysis of R_T0 saliva resulted in the identification of 22 exclusive proteins (Figure 1) with molecular mass in the range of 7.9–158.4 kDa (Table S3). Three of these proteins, namely, S100A2, TPD52, and GOT1, are classified as Cancer Related proteins (in bold). TPD52 and S100A2 are also classified as “Candidate Cancer Biomarker” (underlined).

TPD52 is expressed in several types of normal tissues and has shown altered expression in several cancers, mainly exhibiting overexpression [81]. Recently, human blood expression analysis revealed upregulation of TPD52 in high-grade glioma, moreover increased in the secondary metastatic group of patients with respect to the primary metastatic group [82]. Although the Human Protein Atlas reports TPD52 as not prognostic in glioma, its exclusive finding in R_T0 saliva could ascribe to the protein a potential role as a diagnostic biomarker of tumor recurrence.

Aspartate aminotransferase or Glutamate-Oxaloacetate Transaminase 1 (GOT1) was found rarely expressed or down-expressed in GBM with respect to normal tissues, as demonstrated by a bioinformatics study. Higher levels of the protein are associated with a better prognosis, as confirmed by the enhancement of the malignant tumor phenotype following the knockdown of GOT1 [83]. Alterations of glutamine metabolism are involved in cancer cell survival, proliferation, metastasis, and aggression, with Long Non-Coding RNAs playing a role in modulating the enzymes involved, including GOT1, and contributing to the chemo- and radio-resistance of the tumor [84].

Interestingly, S100A2 is found among the proteins exclusively identified in R_T0 saliva. Elevated expression of S100A2, a protein subjected to epigenetic regulation, was correlated to cell proliferation, apoptosis, invasion, and migration of GBM and associated with poor prognosis [85,86]. Considering the overall S100 proteins identified in the ND_ and R_T0 saliva, different S100 proteins could, therefore, differentiate the ND and R GBM phenotypes in saliva.

Interestingly, GOT1 is included, together with IGKV3, HDGF, and UBC, in the group of proteins previously identified in GBM CUSA fluid [22]. Particularly, GOT1 was exclusively identified in the CUSA fluid of ND and R tumor peripheries, while HDGF and IGKV3 were exclusively identified in the R tumor CORE and in the R A^+^ tumor periphery, respectively. Thus, these two proteins, marking the tumor zone, could have a potential role as biomarkers of tumor recurrence that should be further investigated.

**Figure 7:**
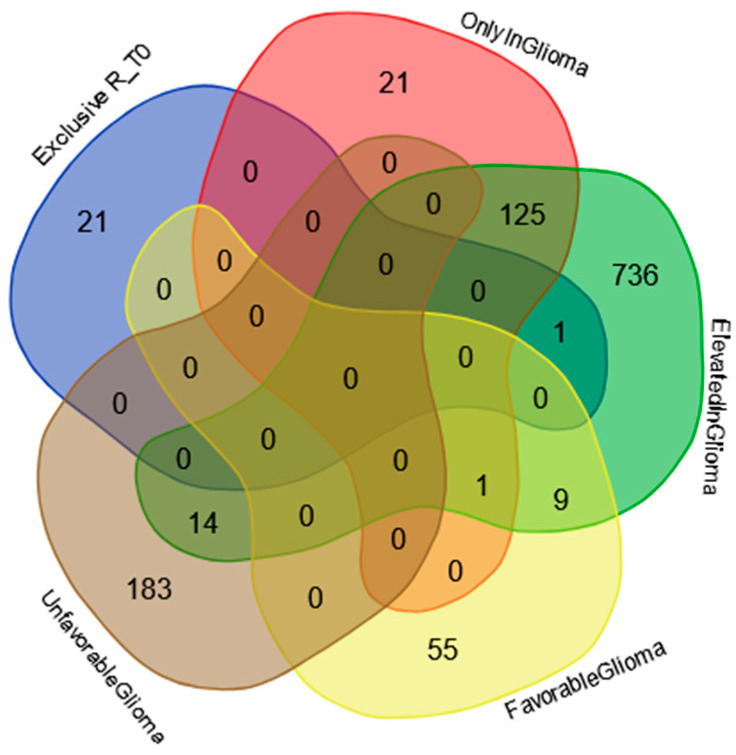
Venn diagram resulting from grouping analysis of the exclusive proteins identified in the R_T0 saliva pool versus different gene categories, namely, “gene elevated expression in glioma”, “gene only detected in glioma”, “unfavorable prognostic genes in GBM”, and “favorable prognostic genes in GBM”, downloaded from the Human Protein Atlas database.

The Venn diagram in Figure 7 shows the distribution of the proteins exclusively identified in the R_T0 saliva (Table S3) among the specific gene classification for glioma pathology reported in The Human Protein Atlas database (https://www.proteinatlas.org/humanproteome/cancer/glioblastoma+multiforme, accessed on 7 August 2024). Among the 22 proteins exclusively identified in R_T0 saliva, only the Homeodomain-only protein (HOPX) was the product of a gene with elevated expression in glioma. Although HOPX is generally considered a tumor suppressor gene that is typically downregulated in various cancers, it has been found to be overexpressed in a small number of cancer cell lines [87]. Interestingly, human GBMs largely lack HOPX expression, and its deregulation has been implicated in the development and progression of the disease [88]. The role of this protein in cancer stimulates further investigation, especially in GBM, where the protein seems to mark tumor relapse with respect to newly diagnosed tumors.

Based on The Human Protein Atlas database, NME2P1 and CHIT1, exclusive of R_T0 saliva, are not classified as proteins expressed in salivary glands. Their finding in saliva seems to be related, therefore, to GBM pathology and relapse. NME2P1 was included in the list of proteins downregulated by temozolomide in U87 GBM-treated cells compared to the control group [89]. CHIT1 belongs to the group of chitinases exhibiting a role in the immune system and in several diseases of an inflammatory basis, including cancer. Other chitinases have been studied in GBM. Particularly, the overexpression of CHI3L2 chitinase has been associated with patient prognosis, and YKL-40 with affecting the treatment efficacy of PI3K/AKT-based pathway inhibitors [90]. To the best of our knowledge, no data on CHIT1 are available, stimulating further investigations. The identification of GBM biomarkers in a biofluid of easy accessibility, such as saliva, is highly in demand for the early diagnosis of tumor relapse during patient follow-up with a low-invasiveness analysis, either for monitoring the effectiveness of treatment or for timely therapeutic interventions.

### 2.4. Label-Free Relative Quantitation of the Proteins Identified in All Saliva Pools

The 101 proteins identified in all saliva samples analyzed (see the Venn diagram in Figure 1) were investigated to evaluate their quantitative variations between the pools by comparing the protein area values of Proteome Discoverer data elaboration through label-free relative quantitation. The quantitative data of the 101 proteins in alphabetical order are summarized in Table S4, where the protein area values of each analytical replicate (*n* = 3) and their relative average value, standard deviation (SD), and relative standard deviation (RSD) data are reported.

Table S5 shows the values of the protein area ratio in the different ND GBM pools compared to CTRL. Additionally, the protein area ratio between ND_ and R_T0 saliva is reported. Statistically significant differences in the areas were evaluated through *t*-test analysis. The first comparison was performed between ND_T0 saliva and CTRL. As can be observed in Table S5, all proteins showed statistically significant different levels in ND_T0 saliva with respect to CTRL saliva, with very few exceptions. The pink color outlines the 28 proteins out of the 101 total with the ND_T0/CTRL ratio value > 10. Among them, Hemoglobin alpha and beta subunits, Mucin-7, 14-3-3 protein zeta/delta, Alpha-2-macroglobulin-like protein 1, Annexins A1 and A2, Calmodulin-like protein 3, Peroxiredoxin-1, Protein LEG1 homolog, Protein S100-A11, and Protein-glutamine gamma-glutamyltransferase E showed the most relevant differences, with increased levels in ND_T0 saliva with respect to CTRL. Alpha-amylase 1B was the unique protein showing a ND_T0/CTRL protein area ratio < 1. The proteins classified as having high expression in salivary glands in the Human Protein Atlas database (marked in grey color in Table S5) generally showed little or no significant variations, with the exception of Carbonic anhydrase 6 (*p*-value **), Mucin-5B (*p*-value ***), Involucrin (*p*-value **), and Polymeric immunoglobulin receptor (*p*-value ***), showing ND_T0/CTRL ratio values > 3.5.

The protein area ratios ND_T1/CTRL, ND_T3/CTRL, R_T0/CTRL, and ND_T0/R_T0 in Table S5 depict a preliminary overview of the quantitative trend of variation of these proteins during the follow-up and tumor relapse. ND_T1 saliva was collected one month after the surgical removal of the tumor; therefore, it could be considered to represent the “healthiest” saliva in the investigation. ND_T3 saliva was instead collected three months after the surgery and radio-chemotherapy combined treatment.

Many proteins exhibited a significant reduction in their levels in ND_T1 and T3 saliva with respect to ND_T0. For example, the ND/CTRL ratio of 14-3-3 zeta/delta protein decreased from 24.58 (ND_T0/CTRL) to 7.36 (ND_T1/CTRL) and then to 5.77 (ND_T3/CTRL), suggesting a response of the protein expression to treatments, either surgery or co-adjuvant therapies. A similar trend was observed for proteins such as Alpha-2-macroglobulin-like protein 1, Annexin A1 and A2, Calmodulin-like protein 3, Cornulin, Endoplasmic reticulum chaperone BiP, Heat shock 70 kDa protein 1A, Heat shock cognate 71 kDa protein, Hemoglobin subunit alpha and beta, Leukocyte elastase inhibitor, Peroxiredoxin 1 and 6, Protein S100-A8, Serine protease inhibitor Kazal-type 5, and Thioredoxin.

Certain proteins, such as Adenylyl cyclase-associated protein 1, Cofilin-1, Peroxiredoxin-5 mitochondrial, Profilin-1, Prosaposin, Protein S100-A9, Vimentin, and Vitamin D-binding protein, instead showed similar levels at the different time of collection of ND GBM saliva.

Alpha-amylase 1B was the unique protein in Table S5 exhibiting a ND_T0/CTRL ratio value <1, thus showing a decrease in tumor saliva with respect to CTRL, which was even more evident in R_T0 saliva. All the other proteins in Table S5 showed higher levels in ND_T0 saliva with respect to CTRL. This was not always recognized in the R_T0/CTRL saliva ratio, which showed a different trend in numerous cases.

The proteins in Table S5 that show significantly different levels and the same trend in ND_ and R_T0 saliva with respect to CTRL and T1 and T3 saliva were graphed to compare the area values in the analytical triplicates and analyzed by one-way ANOVA with Tukey’s post hoc test to disclose potential significant modulations.

**Figure 8:**
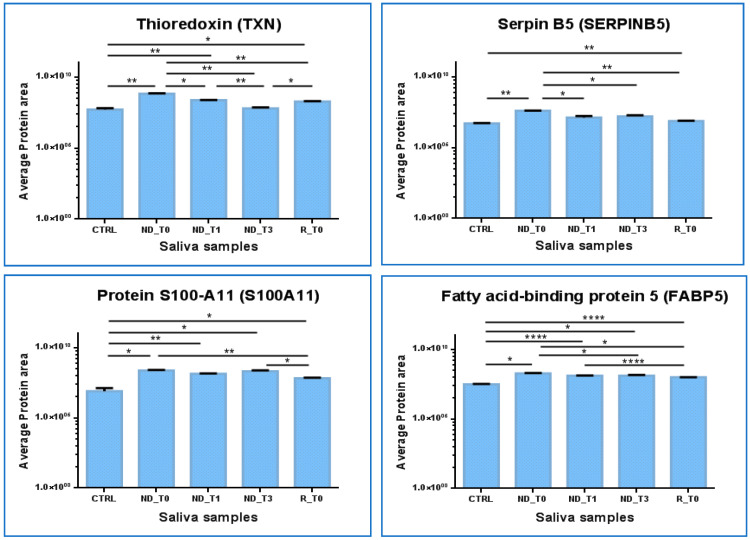
Label-free relative quantitation of TXN, SERPINB5, FABP5, and S100A11 protein area values (in log scale) in CTRL, ND_T0, T1, T3, and R_T0 saliva. Statistical significance was obtained by one-way ANOVA and Tukey’s multiple comparisons test (* *p*-value < 0.05, ** *p*-value < 0.01, **** *p*-value < 0.0001).

The Thioredoxin (TXN) protein results are particularly interesting (Figure 8). TXN showed significantly increased levels in both ND_ and R_T0 pre-surgery saliva with respect to CTRL (ND_T0/CTRL ratio > 10; R_T0/CTRL ratio 3.64, Table S5). Interestingly, the protein showed a response to treatments, either tumor surgical resection or co-adjuvant treatments, revealing a progressive decrease in the level up to values not significantly different from CTRL in T3 saliva. Similar to TXN, SERPINB5 showed increased levels in ND_T0 saliva with respect to CTRL and exhibited a decrease in post-surgery saliva, reaching levels not significantly different from CTRL in both ND_T1 and T3 saliva. The statistically significantly increased area observed in R_T0 saliva compared to CTRL could suggest a potential role of these proteins as diagnostic and prognostic GBM biomarkers to be deeply investigated.

In contrast, Fatty acid binding protein 5 (FABP5) and Protein S100-A11 (S100A11) maintained almost stable higher levels in all tumor saliva pools with respect to CTRL. These proteins could, therefore, have even more potential to mark ND GBM as well as tumor recurrence. Both of these proteins showed statistically different higher levels in R_T0 saliva with respect to CTRL; however, in tumor recurrence, the protein area values were lower than in the ND_T0 pool.

Interestingly, the role of TXN in GBM has already been outlined in the literature, and specific compounds targeting TXN and the thioredoxin reductase system, which is involved in tumor chemoresistance, have been explored as anticancer co-therapy, with mechanisms promoting autophagy [91,92].

The functional interactions of TNX retrieved by the STRING tool evidenced its relationships with Peroxiredoxin 1 (PRDX1), 2 (PRDX2), and 5 (PRDX5). Of note, PRDX2 was included in the present investigation in the group of proteins that exclusively marked the ND_ and R_T0 pre-surgery saliva. PRDX1 and 5, proteins commonly identified in all pools, showed statistically significant higher levels in ND saliva pools with respect to CTRL but not in R_T0 saliva; therefore, they were excluded from the graphical representations in Figure 8.

SerpinB5 was reported as implicated in the survival of GBM cancer stem cells, tumor growth, and resistance to radiation therapy [93].

The role of FABP5 in cancer is an interesting field of research. This protein, involved in fatty-acid transport and metabolism, is generally overexpressed in cancers, including IDH wild-type glioblastoma [94]. In GBM, overexpression of the protein was found to be associated with tumor progression, high-grade histotypes, worse prognosis, and resistance to temozolomide treatment. Accordingly, in ND saliva, we found that the protein level also remained high after treatments.

A recent review on the role of S100 proteins in glioma [95] reported the role of S100A11 in GBM proliferation and invasion and its association with poor prognosis.

These four proteins, identified in CTRL and commonly found in all GBM saliva pools analyzed, showed, in particular, an interesting modulation, which outlines new hints for future investigations.

### 2.5. Protein–Protein Functional Interaction Network of GBM Tumor Salivary Proteins

**Figure 9:**
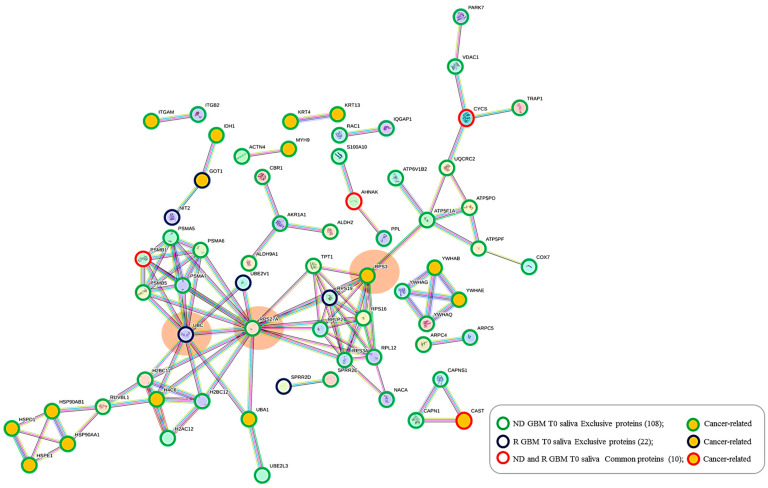
STRING tool protein–protein interaction (PPI) network (highest confidence level 0.900, disconnected nodes hidden) of the 140 salivary proteins identified in ND_ and R_T0 GBM saliva, including their exclusive as well as their shared proteins. The edges represent protein-protein associations, with each color indicating a specific type: Known interactions (light blue: curated databases, purple: experimentally determined); Predicted interactions (green: gene neighborhood, red: gene fusions, blue: gene co-occurrence); Others (ochre: text mining, black: co-expression, light blue: protein homology). In the network, the group of proteins exclusive of ND_ and R-T0 saliva and identified in both, as well as the Cancer Related proteins, are highlighted as described in the legend.

All 140 of the proteins peculiar to tumor saliva, namely, the 108 and 22 proteins exclusive of ND_ and R_T0 saliva, respectively, alongside their 10 common proteins, have been further analyzed by protein–protein interaction (PPI) network for investigating their functional relationships by the STRING tool, particularly highlighting the Cancer Related proteins (Figure 9). It is interesting to observe a dense network of interactions between the different groups of proteins, which include several proteins classified as Cancer Related according to the Human Protein Atlas database. Particularly, three main interacting nodes are evident in the network (circled in orange color), i.e., ubiquitin-ribosomal protein eS31 fusion protein (RPS27A) and small ribosomal subunit protein uS3 (RPS3)—both proteins exclusive of ND_T0 saliva—and polyubiquitin-C (UBC), exclusive of R-T0 saliva. Particularly, the analysis of the network showed RPS27A and RPS3, together with seven other highly interconnected proteins, namely RPS3A, RPL12, RPS16, RPLP2, NACA, RPS19, and TPT1, as constituents of the local STRING network cluster with the long name of “Mixed, incl. Eukaryotic Translation Elongation, and This family consists of several GAGE and XAGE proteins which are found exclusively in humans. The function of this family is unknown although they have been implicated in human cancers”, which is among the most significantly enriched in the network (FDR 0.00099; strength 0.98; count in network 9 of 140), including proteins altered in GBM [96].

UBC and RPS27A are components of the “Constitutive Signaling by Ligand-Responsive EGFR Tumor Variants” reactome pathway that is enriched in the network. The activation of EGFR signaling in cancer commonly occurs as a consequence of an EGFR gene mutation generating oncogenic EGFR mutated forms [97]. EGFR amplification typically occurs in GBM, and EGFR mutations happen in the very background of this process. This pathway also includes the heat shock protein HSP90AA1, exclusive of ND_T0 saliva, which interacts with three other cancer-related proteins (HSP90AB1, HSPE1, and HSPD1), all exclusive of ND_T0 saliva. Interestingly, the proteins UBC, RPS27A, HSP90AA1, and HSP90AB1 are also implicated in the autophagy pathway, together with PARK7 and VDAC1, which are nodes not directly interconnected with them, and an alteration of the autophagy process has been observed in GBM [98,99]. It is noteworthy that the 10 proteins shared between the two groups of saliva T0 (ND-R) maintain in this network their PPI interconnections, although they also show other interactions.

Some of the proteins identified in ND and R tumor saliva deserve a specific mention since they have been previously identified in the extracellular vesicle (EV) fraction of different biofluids, namely, urine, plasma, neurosurgical aspirate, saliva, and GBM cell secretome [33,100,101,102,103], as potential biomarkers. Furthermore, as already discussed and marked in Tables S1–S3 and S5, many proteins identified in saliva have also been identified in GBM CUSA fluid in our previous proteomic investigation [104], which is important in supporting their potential role in disease onset and diffusion. These proteins are specifically HSP90AB1 [22,33,100,102,103,104], HSP90AA1 [22,100,102,103,104], YWHAE [22,33,100,104], PARK7 [22,100,101,104], ATPSF1A [22,100,102,104], MYH9 [22,100,103,104], VDAC1 [22,100,102,104], UQCRC2 [22,100,102,104], CAPZA1 [22,100,102,104], and PSMB5 [100,103], exclusive of ND_T0 saliva; ATRN [100,103], CTSA [100,103], PRDX2 [22,33,104] and PSMB1 [100,103], exclusive of R_T0 saliva; and PRDX2 [22,33,104] and PSMB1 [100,103], shared in the exclusive list of ND and R_T0 saliva.

It is noteworthy that these studies also reported the characterization of some of the proteins identified in all saliva pools, namely, PSAPs [22,100,101,103,104], FABP5 [22,33,100,102,104], ALDOA [22,33,100,103,104], CSTB [22,100,102,104], ANXA1 [22,100,102,103,104], TPI1 [22,100,103,104], S100A11 [22,100,102,104], ANXA2 [22,100,102,104], and CTSD [22,100,103,104], some of them exhibiting statistically significant different levels between the pools, specifically the FABP5, in the present investigation.

## 3. Materials and Methods

### 3.1. Sample Collection

The study received approval from the Ethical Committee of the Catholic University of Rome under reference number 13891/18 ID 2015; date of approval: 18 May 2018. Saliva samples were obtained from patients affected by IDH-wt GBM, specifically from three patients with newly diagnosed (ND) GBM (mean age 52.3 ± 6.43 years) and three patients with relapsed (R) GBM (mean age 48.6 ± 8.1 years), after receiving written consent. Additionally, control (CTRL) saliva samples were collected from three healthy donors (mean age 52.3 ± 4.6 years). Saliva samples (approximately 0.3 mL) were collected from the same patient before surgery (T0) in cases of newly diagnosed and relapsed glioblastoma and during the follow-up, specifically one month after surgery before treatments (T1) and three months after surgery, following radiotherapy combined with temozolomide chemotherapy (T3). Whole saliva samples were collected between 11:00 a.m. and 12:00 p.m., fasting and without any stimulation, after rinsing the oral cavity. The saliva was taken from the anterior floor of the mouth of the donors using a sterile disposable Pasteur pipette, and then it was transferred to a plastic tube and processed within 30 min of collection. Each sample was diluted 1:1 (*v*/*v*) with a 0.2% (*v*/*v*) aqueous formic acid solution and then centrifuged at 14,000 rpm for 5 min at 4 °C. The acid-soluble supernatant was collected and stored at −80 °C until further analysis.

### 3.2. Chemicals

Formic acid (FA), water, acetonitrile (ACN), and centrifugal filter units Microcon 10 were from Merck (Darmstadt, Germany). Urea, Tris (hydroxymethyl) aminomethane, D,L-dithiothreitol (DTT), ammonium bicarbonate (AMBIC), Iodoacetamide (IAA), Protease Inhibitor Cocktail (PIC), and bovine serum albumin were from Sigma-Aldrich (St. Louis, MO, USA). Trypsin enzyme (Trypsin Gold, Mass Spectrometry Grade) was from Promega Corporation (Madison, WI, USA).

### 3.3. Sample Preparation

To establish the total protein concentration of each collected saliva sample, the Bradford Assay (Bio-Rad Laboratories, Hercules, CA, USA) was employed with UV–Visible spectrophotometry (8453 UV-Vis Supplies, Agilent Technologies, Waldbronn, Germany) using bovine serum albumin as a reference standard. The total protein content was determined as follows: the mean values were 0.57 ± 0.41 μg/μL, 0.51 ± 0.38 μg/μL, and 0.95 ± 0.05 μg/μL for saliva samples collected at T0, T1, and T3, respectively. For the relapse T0 saliva sample, the mean value was 0.51 ± 0.38 μg/μL. Additionally, control (CTRL) saliva samples from healthy donors had a mean value of 0.57 ± 0.53 μg/μL. Saliva samples from patients with GBM were grouped based on collection time, namely T0, T1, and T3, and combined volumes of each individual sample containing an equal quantity of total proteins, i.e., 50 μg. The T0, T1, and T3 pools had final concentrations of 0.37, 0.31, and 0.94 μg/μL, respectively. Regarding saliva samples from patients with recurrent GBM, volumes of each individual sample containing the same quantity of total proteins, i.e., 30 μg, were pooled. The T0 pool had a final concentration of 0.344 μg/μL in the recurrent sample. The control saliva pool had a mean concentration of 0.27 μg/μL. The Filter Aided Sample Preparation (FASP) protocol was employed to process saliva samples for proteomic analysis, as previously described [105], loading a total protein content in the range of 30–50 µg diluted up to a volume of 200 µL with a 0.1% (*v*/*v*) aqueous formic acid solution before transferring into the 10 kDa cut-off centrifugal filter devices. After centrifugation, the filter was washed three times with urea buffer solution containing Tris buffer100 mM and urea 8 M pH 8.5 to ensure efficient buffer exchange. A solution of D,L-dithiothreitol (DTT) was then added, and the sample was incubated for 15 min at 37 °C to reduce the disulfide bonds of the proteins. Subsequently, a solution of iodoacetamide (IAA) was added and incubated for 20 min at room temperature in the dark to alkylate the reduced proteins. Excess IAA was removed by adding a DTT solution. The filter was then washed with AMBIC solution to facilitate buffer exchange for enzymatic digestion with trypsin. The trypsin solution was added to the filter (total protein content: enzyme 1:50, *w*/*w*), and the proteins were digested overnight at 37 °C. Finally, the filtrate was acidified with 10% formic acid final concentration (*v*/*v*) to stop the reaction and stored at −80 °C after lyophilization. Before LC-MS analysis, the samples were reconstituted in 0.1% (*v*/*v*) formic acid solution with a final concentration of 1 μg/μL.

### 3.4. LC-MS Proteomic Analysis

LC-ESI-MS/MS analyses were performed in duplicate on an UltiMate 3000 RSLCnano System coupled to an Orbitrap Elite MS detector with an EASY-Spray nanoESI source (Thermo Fisher Scientific, Waltham, MA, USA) and the Thermo Xcalibur 2.2 computer program (Thermo Fisher Scientific) for instrumental operation and data acquisition. Chromatographic separation was performed on an EASY-Spray PepMap C18 column (15 cm in length × 50 μm of internal diameter (ID), 2 μm particles, 100 Å pore size) (Thermo Fisher Scientific) coupled to an Acclaim PepMap100 nano-trap cartridge (C18, 5 μm, 100 Å, 300 μm i.d. × 5 mm) (Thermo Fisher Scientific). Separation was performed at 40 °C in gradient elution at a mobile phase flow rate of 0.3 μL/min using aqueous FA solution (0.1%, *v*/*v*) as eluent A and ACN/FA solution (99.9:0.1, *v*/*v*) as eluent B, as follows: (i) 5% B (7 min), (ii) from 5% to 35% B (113 min), (iii) from 35% B to 99% (2 min), (iv) 99% B (3 min), (v) from 99% to 1.6% B (2 min), (vi) 1.6% B (3 min), (vii) from 1.6% to 78% B (3 min), (viii) 78% B (3 min), (ix) from 78% to 1.6% B (3 min), (x) 1.6% B (3 min), (xi) from 1.6% to 78% B (3 min), (xii) 78% B (3 min), (xiii) from 78% B to 5% B (2 min), and (xiv) 5% B (20 min). The injection volume was 5 μL. The Orbitrap Elite instrument was operated in positive ionization mode at a 60,000 full scan resolution in 350–2000 *m*/*z* acquisition range, performing MS/MS fragmentation by collision-induced dissociation (CID, 35% normalized collision energy) of the 20 most intense signals of each MS spectrum in Data-Dependent Scan (DDS) mode. The minimum signal was set to 500.0, the isolation width to 2 *m*/*z,* and the default charge state to +2. MS/MS spectra acquisition was performed in the linear ion trap at a normal scan rate. CTRL saliva pool analyses were performed by a UHPLC-MS/MS UltiMate 3000 RSLCnano System coupled to an Orbitrap Fusion Lumos Tribrid Mass Spectrometer and EASY-Spray nanoESI (Thermo Fisher Scientific, Waltham, MA, USA) following the same chromatographic and mass spectrometer operating conditions as described above.

### 3.5. LC-MS Data Elaboration and Bioinformatic Analysis

LC-MS and MS/MS data were elaborated by Proteome Discoverer software (version 1.4.1.14, Thermo Fisher Scientific), based on the SEQUEST HT cluster as the search engine against the Swiss-Prot Homo Sapiens proteome (UniProtKb, Swiss-Prot, homo+sapiens released in February 2024) by applying the following spectrum filters: minimum precursor mass 350 Da, maximum precursor mass 10,000 Da, total intensity threshold 0.0, and minimum peak count 1. The signal-to-noise (S/N) threshold was set to 1.5. The set enzyme was trypsin with a maximum of 2 missed cleavage sites; minimum and maximum peptide lengths were 6 and 144 residues, respectively. Tolerance tools were mass tolerance 10 ppm; fragment mass tolerances 0.5 Da and 0.02 Da; use average precursor mass False; and use average fragment mass False. The set dynamic modifications were methionine oxidation (+15.99 Da) and acetylation N-Terminus (+42.011 Da); carbamidomethylation of cysteine (+57.02 Da) was the static modification set. Protein and peptide identification was validated by the Percolator node, with the strict target value of FDR set at 0.01 and the relaxed value at 0.05. Protein identification results were filtered according to the Human Proteome Project (HPP) Mass Spectrometry Data Interpretation Guidelines [18]: high confidence, minimum peptide length ≥ 9 amino acids, peptide rank 1, and minimum two peptides with these features for protein.

Label-free proteins‘ relative quantitation was performed on protein area values obtained by LC-MS data elaboration by Proteome Discoverer software, and significant differences were evaluated by one-way ANOVA with Tukey’s post hoc test on analytical triplicates. A Venn diagram tool [106] was used to highlight common and selective proteins in the pools, while the STRING tool was used to predict protein–protein functional interactions [107]. Selected classification of proteins was performed using The Human Protein Atlas database as a reference [19,20,21].

## 4. Conclusions

In this study, we demonstrated that saliva showed great potential as a biological matrix to investigate GBM biomarkers, and it also features minimally invasive collection and high patient compliance. The results of the present investigation are supported by literature data since several of the identified salivary proteins have been characterized in other GBM biofluids and, specifically in their extracellular vesicle fractions, some of them having a role as potential biomarkers. Furthermore, the identification of selected salivary proteins in CUSA GBM fluid was an important finding that supports their potential role in the disease as diagnostic or prognostic biomarkers.

In conclusion, ten proteins (CYCS, PRDX2, RAB1C, PSMB1, KLK6, TMOD3, PAI2, PLBD1, CAST, and AHNAK) depict the GBM core salivary protein profile, marking either the newly diagnosed tumor or the tumor relapse. Particularly, the identification in CUSA fluid [22] of PRDX2 and RAB1C, together with PADI4 and CRYAB, which are included in the most abundant proteins exclusively identified in ND_T0 saliva, reinforces the need to deeply investigate these proteins as potential GBM biomarkers in saliva. TPD52, exclusively identified in the R_T0 saliva, was reported as upregulated in the human plasma of high-grade glioma patients and associated with a secondary metastatic group of patients; it should instead be further investigated as a potential biomarker of recurrence.

In conclusion, our results confirmed the potential of saliva biofluid in enabling the identification of potential disease biomarkers that could help in the diagnosis of glioblastoma in its early stage, when treatment options may be more effective.

Periodic monitoring of tumor biomarkers in saliva could be a useful tool for assessing response to therapy and detecting tumor recurrence in a timely manner, with minimally invasive sampling and high patient compliance. The results of this pilot investigation provide new insights into the molecular profile of GBM saliva, outlining a panel of potential protein biomarkers to be investigated more thoroughly with validation assays in view of the development of potential diagnostic/prognostic clinical applications in the context of precision medicine.

## Figures and Tables

**Table 1:** List of the 10 proteins exclusively identified in ND_ and R_T0 saliva pools.

Accession *	Description ^#§^	Gene Name	kDa	CUSA ND CORE	CUSA ND A+	CUSA ND A−	CUSA R CORE	CUSA R A+	CUSA R A−
P99999 *	Cytochrome c	*CYCS*	11.7	-	x	-	-	x	-
P32119 *	**Peroxiredoxin-2**	***PRDX2***	**21.9**	x	x	x	x	x	x
Q92928 *	Putative Ras-related protein Rab-1C	*RAB1C*	22.0	-	x	-	-	-	-
P20618	Proteasome subunit beta type-1	*PSMB1*	26.5	-	-	-	-	-	-
**Q92876**	**Kallikrein-6 **	***KLK6***	**26.8**	-	-	-	-	-	-
Q9NYL9	Tropomodulin-3	*TMOD3*	39.6	-	-	-	-	-	-
**P05120**	**Plasminogen activator inhibitor 2**	***(PAI2)***	**46.6**	-	-	-	-	-	-
Q6P4A8	Phospholipase B-like 1	*PLBD1*	6.3	-	-	-	-	-	-
P20810 *	**Calpastatin**	*CAST*	76.5	-	-	x	-	x	-
Q09666 *	Neuroblast differentiation-associated protein AHNAK	*AHNAK*	62.9	-	-	-	**x**	-	-

* Proteins identified in GBM CUSA fluid [22]. # Proteins classified as Cancer Related are marked in bold. § Proteins classified as Candidate Cancer Biomarkers in the Human Protein Atlas database are underlined (x, identified protein; -, not identified).

## Data Availability

Data are contained within the article or Supplementary Material.

## Supplementary Materials

The following supporting information can be downloaded at: https://www.mdpi.com/article/10.3390/ijms252312984/s1.
