# Supplementary material for: Proteomic Profiling of Pre- and Post-Surgery Saliva of Glioblastoma Patients: A Pilot Investigation
Source: Int J Mol Sci. 2024 Dec 3;25(23):12984. doi: 10.3390/ijms252312984 (PMC11641718; doi:10.3390/ijms252312984)
Supplement: Supplementary file 1 [file ijms-25-12984-s001.zip › Table S5.pdf]

Table S5. ND_T0, ND_T1, ND_T3, R_T0/CTRL area ratio for the 101 salivary proteins identified in all pools. The ratio ND_T0/R_T0 is also reported. Statistical significance was assessed using the t-test p-value.

Accession	Description	Gene name	ND_T0/ CTRL[#] (p-value)[§]	ND_T1/ CTRL[#] (p-value)[§]	ND_T3/ CTRL[#] (p-value)[§]	R_T0/ CTRL[#] (p-value)[§]	ND_T0/ R_T0[#] (p-value)[§]
P63104	14-3-3 protein zeta/delta	YWHAZ	24.58 (***)	7.36 (**)	5.77 (**)	0.80 (-)	30.63 (***)
P60709	Actin, cytoplasmic 1	ACTB	9.22 (***)	6.47 (***)	4.06 (***)	0.24 (**)	38.63 (***)
Q01518	Adenylyl cyclase-associated protein 1	CAP1	7.61 (***)	8.23 (***)	8.22 (***)	0.74 (-)	10.24 (***)
P02768	Albumin	ALB	3.04 (**)	5.67 (***)	7.56 (***)	0.46 (***)	6.61 (***)
P0DTE7	Alpha-amylase 1B	AMY1B	0.70 (*)	0.64 (**)	0.42 (**)	0.13 (***)	0.57 (***)
A8K2U0	Alpha-2-macroglobulin-like protein 1	A2ML1	26.02 (***)	3.69 (**)	2.91 (***)	1.22 (**)	21.38 (***)
P06733	Alpha-enolase	ENO1	6.57 (***)	2.45 (**)	3.00 (***)	0.77 (-)	8.54 (***)
P04083	Annexin A1	ANXA1	22.43 (***)	3.31 (***)	5.44 (***)	2.35 (***)	9.53 (***)
P07355	Annexin A2	ANXA2	36.57 (***)	7.07 (***)	7.67 (***)	3.45 (***)	10.60 (***)
P12429	Annexin A3	ANXA3	4.00 (**)	2.32 (***)	4.40 (***)	1.26 (-)	3.17 (**)
P02647	Apolipoprotein A-I	APOA1	3.57 (**)	8.62 (***)	9.51 (***)	0.23 (***)	15.76 (**)
Q96DR5	BPI fold-containing family A member 2	BPIFA2	0.99 (-)	0.47 (**)	0.20 (***)	0.12 (**)	8.07 (***)
P80723	Brain acid soluble protein 1	BASP1	2.42 (-)	10.31 (-)	8.62 (**)	0.72 (-)	3.37 (**)
P27482	Calmodulin-like protein 3	CALML3	20.89 (***)	4.78 (***)	3.63 (-)	0.94 (-)	22.22 (***)
P23280	Carbonic anhydrase 6	CA6	3.92 (***)	3.15 (***)	2.47 (**)	0.87 (**)	4.48 (***)
P07339	Cathepsin D	CTSD	2.33 (***)	1.95 (-)	1.43 (*)	1.39 (-)	1.68 (***)
P00450	Ceruloplasmin	CP	2.00 (***)	1.21 (-)	1.92 (**)	0.36 (**)	5.55 (***)
P23528	Cofilin-1	CFL1	7.64 (**)	5.87 (*)	7.63 (**)	0.97 (-)	7.85 (*)
P01024	Complement C3	C3	11.92 (***)	16.55 (***)	19.69 (***)	0.61 (-)	19.41 (***)
P35321	Cornifin-A	SPRR1A	5.58 (**)	7.83 (***)	4.61 (***)	2.60 (**)	2.15 (**)
Q9UBG3	Cornulin	CRNN	11.49 (***)	2.90 (**)	1.06 (-)	1.42 (**)	8.12 (***)
P31146	Coronin-1A	CORO1A	17.14 (***)	15.57 (*)	32.85 (***)	3.08 (-)	5.57 (**)
P04080	Cystatin-B	CSTB	9.26 (***)	1.89 (**)	0.51 (**)	1.25 (-)	7.39 (***)
P01034	Cystatin-C	CYTC	1.10 (-)	0.42 (**)	0.45 (*)	0.44 (-)	0.42 (**)
P01036	Cystatin-S	CST4	1.59 (-)	1.86 (-)	0.80 (-)	1.35 (-)	1.18 (-)
P09228	Cystatin-SA	CST2	2.49 (-)	3.30 (**)	1.49 (-)	1.89 (-)	1.32 (-)
P01037	Cystatin-SN	CST1	1.86 (-)	2.17 (*)	0.94 (-)	1.58 (**)	1.18 (-)
P54108	Cysteine-rich secretory protein 3	CRISP3	1.76 (***)	2.95 (**)	3.14 (**)	1.11 (-)	1.59 (***)
Q9UGM3	Deleted in malignant brain tumors 1 protein	DMBT1	2.62 (**)	1.26 (**)	1.33 (-)	0.92 (-)	2.85 (**)
Q02487	Desmocollin-2	DSC2	5.41 (***)	1.39 (**)	1.24 (-)	1.15 (-)	4.70 (***)
Q02413	Desmoglein-1	DSG1	7.29 (***)	0.86 (-)	0.14 (**)	0.82 (-)	8.93 (***)
P32926	Desmoglein-3	DSG3	9.81 (***)	5.22 (***)	2.24 (-)	1.71 (**)	5.75 (***)
P11021	Endoplasmic reticulum chaperone BiP	HSPA5	15.56 (***)	3.55 (-)	3.61 (**)	0.33 (***)	47.58 (***)
Q01469	Fatty acid-binding protein 5	FABP5	6.15 (***)	4.22 (***)	4.22 (***)	3.19 (***)	1.93 (***)
P02675	Fibrinogen beta chain	FGB	3.37 (**)	4.66 (***)	6.43 (***)	0.31 (***)	10.97 (***)
P04075	Fructose-bisphosphate aldolase A	ALDOA	34.95 (***)	15.35 (***)	20.27 (***)	2.18 (**)	16.06 (***)
P06396	Gelsolin	GSN	7.36 (***)	7.81 (***)	11.54 (***)	0.36 (**)	20.23 (***)
P04406	Glyceraldehyde-3-phosphate dehydrogenase	GAPDH	6.68 (***)	1.26 (-)	0.97 (-)	0.16 (**)	41.29 (***)
P00738	Haptoglobin	HP	2.61 (***)	3.33 (***)	8.70 (***)	0.24 (***)	10.82 (***)
P0DMV8	Heat shock 70 kDa protein 1A	HSPA1A	11.65 (***)	2.86 (*)	3.20 (***)	0.15 (**)	79.66 (***)
P11142	Heat shock cognate 71 kDa protein	HSPA8	13.62 (***)	3.14 (*)	3.37 (***)	0.26 (***)	52.05 (***)
P69905	Hemoglobin subunit alpha	HBA1	2938.48 (***)	349.90 (***)	834.16 (***)	6.63 (***)	443.31 (***)

P68871	Hemoglobin subunit beta	HBB	4183.40 (***)	564.60 (***)	829.18 (***)	9.67 (***)	432.77 (***)
P02790	Hemopexin	HPX	1.71 (*)	1.42 (***)	1.16 (-)	0.42 (**)	4.06 (***)
P0DOX2	Immunoglobulin alpha-2	IGA2	1.11 (-)	0.33 (**)	0.57 (-)	0.59 (**)	1.87 (*)
P01876	Immunoglobulin heavy constant alpha 1	IGHA1	2.79 (**)	1.02 (-)	2.23 (*)	0.44 (**)	6.31 (***)
P01764	Immunoglobulin heavy variable 3-23 OS	IGHV3-23	6.21 (***)	2.33 (***)	3.23 (**)	0.87 (-)	7.16 (***)
P01591	Immunoglobulin J chain	JCHAIN	2.91 (**)	0.84 (-)	1.62 (**)	0.50 (**)	5.82 (***)
P01834	Immunoglobulin kappa constant	IGKC	4.83 (***)	2.21 (**)	3.26 (***)	0.62 (**)	7.77 (***)
P07476	Involucrin	IVL	5.34 (**)	1.96 (**)	0.68 (**)	0.65 (**)	8.17 (***)
P06870	Kallikrein-1	KLK1	3.59 (*)	1.31 (-)	0.99 (-)	1.15 (-)	3.13 (**)
P13645	Keratin, type I cytoskeletal 10	KRT10	5.22 (**)	2.19 (***)	2.68 (***)	0.39 (***)	13.25 (**)
P02533	Keratin, type I cytoskeletal 14	KRT14	5.46 (***)	0.58 (*)	0.63 (-)	0.28 (**)	19.72 (***)
P35527	Keratin, type I cytoskeletal 9	KRT9	1.02 (-)	1.36 (*)	1.99 (***)	0.21 (***)	4.90 (***)
P04264	Keratin, type II cytoskeletal 1	KRT1	2.63 (**)	1.71 (**)	2.04 (**)	0.37 (**)	7.12 (***)
P35908	Keratin, type II cytoskeletal 2 epidermal	KRT2	4.02 (***)	1.77 (**)	2.25 (***)	0.34 (***)	11.66 (***)
P13647	Keratin, type II cytoskeletal 5	KRT5	3.23 (***)	0.91 (-)	0.76 (*)	0.87 (-)	3.70 (**)
P22079	Lactoperoxidase	LPO	1.70 (*)	0.98 (-)	0.73 (-)	0.37 (**)	4.58 (***)
P02788	Lactotransferrin	LTF	3.64 (**)	1.38 (***)	1.22 (-)	0.34 (***)	10.60 (**)
P30740	Leukocyte elastase inhibitor	SERPINB1	12.60 (***)	2.53 (-)	1.39 (**)	0.63 (-)	20.02 (***)
O95274	Ly6/PLAUR domain-containing protein 3	LYPD3	13.77 (***)	14.37 (***)	10.70 (***)	6.03 (***)	2.29 (***)
P40926	Malate dehydrogenase, mitochondrial	MDH2	79.97 (***)	10.37 (-)	10.35 (-)	13.51 (**)	5.92 (***)
P14780	Matrix metalloproteinase-9	MMP9	4.09 (*)	9.24 (***)	14.36 (***)	0.65 (***)	6.31 (*)
Q9HC84	Mucin-5B	MUC5B	9.08 (***)	4.42 (***)	16.45 (***)	1.16 (-)	7.80 (***)
Q8TAX7	Mucin-7	MUC7	216.72 (***)	270.95 (**)	159.01 (**)	44.50 (***)	4.87 (***)
P05164	Myeloperoxidase	MPO	15.14 (***)	6.38 (***)	9.09 (***)	0.82 (-)	18.38 (***)
P59665	Neutrophil defensin 1	DEFA1	4.82 (***)	6.24 (***)	8.51 (***)	0.57 (-)	8.47 (***)
P80188	Neutrophil gelatinase-associated lipocalin	LCN2	0.94 (-)	1.21 (-)	0.93 (-)	0.74 (-)	1.27 (*)
Q96DA0	Pancreatic adenocarcinoma up-regulated factor	ZG16B	3.01 (*)	0.78 (-)	0.16 (-)	1.00 (-)	3.01 (***)
O75594	Peptidoglycan recognition protein 1	PGLYRP1	1.93 (-)	2.34 (*)	2.50 (**)	0.73 (-)	2.64 (**)
P62937	Peptidyl-prolyl cis-trans isomerase A	PPIA	3.18 (*)	1.34 (-)	1.59 (-)	0.78 (-)	4.09 (**)
Q06830	Peroxiredoxin-1	PRDX1	21.24 (***)	7.58 (***)	9.33 (***)	3.14 (**)	6.77 (***)
P30044	Peroxiredoxin-5, mitochondrial	PRDX5	4.67 (***)	5.08 (***)	5.56 (**)	2.09 (-)	2.23 (***)
P30041	Peroxiredoxin-6	PRDX6	12.25 (***)	1.21 (-)	0.86 (-)	0.30 (**)	41.35 (***)
P30086	Phosphatidylethanolamine-binding protein 1	PEBP1	5.86 (***)	1.66 (***)	1.48 (-)	1.25 (-)	4.70 (***)
P13796	Plastin-2	LCP1	4.29 (***)	8.67 (***)	12.45 (***)	0.64 (**)	6.70 (***)
P01833	Polymeric immunoglobulin receptor	PIGR	4.89 (**)	1.97 (**)	4.24 (***)	0.86 (-)	5.69 (***)
P07737	Profilin-1	PFN1	1.43 (*)	1.29 (***)	1.26 (*)	0.32 (**)	4.44 (***)
P12273	Prolactin-inducible protein	PIP	2.18 (***)	1.65 (-)	1.10 (**)	0.81 (**)	2.70 (***)
P07602	Prosaposin	PSAP	17.39 (**)	17.62 (**)	17.36 (*)	3.68 (***)	4.72 (*)
Q6P5S2	Protein LEG1 homolog	LEG1	37.43 (**)	64.98 (***)	25.53 (***)	20.60 (***)	1.82 (*)
P31949	Protein S100-A11	S100A11	29.96 (***)	17.20 (***)	26.48 (**)	8.52 (**)	3.52 (***)
P05109	Protein S100-A8	S100A8	10.73 (***)	5.29 (***)	6.55 (***)	1.13 (-)	9.51 (***)
P06702	Protein S100-A9	S100A9	4.96 (***)	3.90 (***)	3.88 (***)	1.28 (**)	3.87 (***)
Q08188	Protein-glutamine gamma-glutamyltransferase E	TGM3	41.67 (***)	7.58 (***)	13.18 (***)	1.96 (***)	21.24 (***)
P52565	Rho GDP-dissociation inhibitor 1	ARHGDIA	2.01 (*)	1.29 (**)	1.44 (***)	0.38 (***)	5.25 (**)
P52566	Rho GDP-dissociation inhibitor 2	ARHGDIB	1.95 (**)	3.35 (**)	4.09 (**)	0.19 (**)	10.06 (***)

Q9NQ38	Serine protease inhibitor Kazal-type 5	SPINK5	12.61 (***)	5.22 (**)	5.30 (**)	3.51 (***)	3.59 (***)
P02787	Serotransferrin	TF	4.07 (***)	2.65 (***)	3.06 (***)	0.48 (**)	8.43 (***)
Q9UIV8	Serpin B13	SERPINB13	9.07 (**)	2.40 (**)	2.34 (**)	2.29 (***)	3.96 (**)
P29508	Serpin B3	SERPINB3	8.76 (*)	3.88 (***)	2.47 (-)	1.59 (***)	5.50 (*)
P36952	Serpin B5	SERPINB5	7.92 (***)	2.60 (*)	3.22 (**)	1.52 (***)	5.21 (***)
Q9UBC9	Small proline-rich protein 3	SPRR3	2.45 (**)	1.78 (**)	0.69 (-)	1.64 (-)	1.50 (**)
P10599	Thioredoxin	TXN	12.87 (***)	4.61 (***)	1.20 (-)	3.64 (**)	3.54 (***)
P37837	Transaldolase	TALDO1	4.13 (***)	3.43 (***)	5.61 (**)	0.68 (-)	6.07 (***)
P29401	Transketolase	TKT	4.61 (**)	1.41 (-)	0.50 (*)	0.22 (**)	21.27 (***)
P60174	Triosephosphate isomerase	TPI1	6.24 (***)	2.80 (***)	2.09 (***)	0.99 (-)	6.29 (***)
P08670	Vimentin	VIM	6.70 (**)	6.74 (*)	8.42 (**)	0.99 (-)	6.80 (-)
P02774	Vitamin D-binding protein	GC	2.22 (*)	2.54 (**)	2.46 (**)	0.43 (-)	5.22 (***)
Q14508	WAP four-disulfide core domain protein 2	WFDC2	2.24 (-)	1.68 (-)	3.08 (-)	1.40 (**)	1.60 (-)
P25311	Zinc-alpha-2-glycoprotein	AZGP1	3.00 (***)	1.69 (***)	2.25 (***)	0.82 (***)	3.65 (***)

§ p-value <0.01 (***), p-value <0.05 (**), p-value <0.1 (*), not significant (-), calculated by t-test on protein area values (values in Table S4).

# the pink color marks the ND-T0/CTRL proteins area ratio > 10; the grey color evidences the proteins with elevated expression in saliva
