# Supplementary material for: Proteomic Profiling of Pre- and Post-Surgery Saliva of Glioblastoma Patients: A Pilot Investigation
Source: Int J Mol Sci. 2024 Dec 3;25(23):12984. doi: 10.3390/ijms252312984 (PMC11641718; doi:10.3390/ijms252312984)
Supplement: Supplementary file 1 [file ijms-25-12984-s001.zip › Table S2.pdf]

Table S2. List of the 108 proteins exclusive of ND_T0 saliva pool. Proteins are listed in increasing order of molecular mass (kDa). Protein identification data in CUSA fluid from different zones, namely tumor CORE and 5-aminolevulinic induced fluorescence positive (A+) and negative (A-) peripheral zones, are also included [22].

Accession*	Description#§£	Gene name	kDa	CUSA ND CORE	CUSA ND A+	CUSA ND A-	CUSA R CORE	CUSA R A+	CUSA R A-
P22531	Small proline-rich protein 2E	SPRR2E	7.8	-	-	-	-	-	-
P14406	Cytochrome c oxidase subunit 7A2, mitochondrial	COX7A2	9.4	-	-	-	-	-	-
**P61604***	**10 kDa heat shock protein, mitochondrial**	**HSPE1**	**10.9**	x	x	x	x	x	x
P60903	Protein S100-A10	S10AA	11.2	-	-	-	-	-	-
**P62805***	**Histone H4**	**H4C1**	**11.4**	-	x	-	-	x	-
Q9HCY8*	Protein S100-A14	S100A14	11.7	-	-	-	-	-	-
P05387*	Large ribosomal subunit protein P2	RPLP2	11.7	-	x	x	x	x	-
P18859*	ATP synthase-coupling factor 6, mitochondrial	ATP5PF	12.6	-	x	x	x	-	x
P01825	Immunoglobulin heavy variable 4-59	IGHV4-59	12.9	-	-	-	-	-	-
P01782	Immunoglobulin heavy variable 3-9	IGHV3-9	12.9	-	-	-	-	-	-
O60814*	Histone H2B type 1-K	H2BC12	13.9	-	x	-	-	x	x
P06899	Histone H2B type 1-J	H2BC11	13.9	-	-	-	-	-	-
Q96KK5*	Histone H2A type 1-H	H2AC12	13.9	x	x	x	x	x	x
P47929	Galectin-7	LGALS7	15.1	-	-	-	-	-	-
P69891*	Hemoglobin subunit gamma-1	HBG1	16.1	-	-	x	x	x	x
O15511	Actin-related protein 2/3 complex subunit 5	ARPC5	16.3	-	-	-	-	-	-
P62249	Small ribosomal subunit protein uS9	RPS16	16.4	-	-	-	-	-	-
P34096	Ribonuclease 4	RNASE4	16.8	-	-	-	-	-	-
**P15531***	**Nucleoside diphosphate kinase A**	**NME1**	**17.1**	-	x	-	-	x	x
Q9UHA7	Interleukin-36 alpha	IL36A	17.7	-	-	-	-	-	-
P30050*	Large ribosomal subunit protein uL11	RPL12	17.8	-	x	-	-	-	-
P68036*	Ubiquitin-conjugating enzyme E2 L3	UBE2L3	17.9	-	x	-	-	-	-
P62979	Ubiquitin-ribosomal protein eS31 fusion protein	RPS27A	18.0	-	-	-	x	-	-
P13693	Translationally-controlled tumor protein	TPT1	19.6	-	-	-	-	-	-
P59998	Actin-related protein 2/3 complex subunit 4	ARPC4	19.6	-	-	-	-	-	-
Q99497 *	Parkinson disease protein 7	PARK7	19.9	x	x	x	x	x	x
**P02511***	**Alpha-crystallin B chain**	**CRYAB**	**20.1**	x	x	x	x	x	x
**P63000***	**Ras-related C3 botulinum toxin substrate 1**	**RAC1**	**21.4**	-	-	-	x	-	x
**P61586***	**Transforming protein RhoA**	**RHOA**	**21.8**	-	x	-	-	x	x
P30085*	UMP-CMP kinase	CMPK1	22.2	-	-	x	x	x	x
P48047	ATP synthase subunit O, mitochondrial	ATP5PO	23.3	-	-	-	-	-	-
Q13765*	Nascent polypeptide-associated complex subunit alpha	NACA	23.4	-	x	-	-	-	-
P28676	Grancalcin	GCA	24.0	-	-	-	-	-	-
P09497	Clathrin light chain B	CLTB	25.2	-	-	-	x	-	-
P22352	Glutathione peroxidase 3	GPX3	25.5	-	-	-	-	-	-
P28066*	Proteasome subunit alpha type-5	PSMA5	26.4	-	x	-	-	x	x
**P23396**	**Small ribosomal subunit protein uS3**	**RPS3**	**26.7**	-	-	-	-	-	-
Q9NP55	BPI fold-containing family A member 1	BPIFA1	26.7	-	-	-	-	-	-

P60900	Proteasome subunit alpha type-6	PSMA6	27.4	-	-	-	-	-	-
P78417	Glutathione S-transferase omega-1	GSTO1	27.5	-	-	-	-	-	-
P30048	Thioredoxin-dependent peroxide reductase, mitochondrial	PRDX3	27.7	-	-	-	-	-	-
P27348 *	14-3-3 protein theta	YWHAQ	27.7	-	x	x	x	x	x
O14818	Proteasome subunit alpha type-7	PSMA7	27.9	-	-	-	-	-	-
**P31946 ***	**14-3-3 protein beta/alpha**	**YWHAB**	**28.1**	x	x	x	x	x	x
P61981 *	14-3-3 protein gamma	YWHAG	28.3	-	x	-	x	x	x
P04632 *	Calpain small subunit 1	CAPNS1	28.3	-	-	-	-	-	x
P28074	Proteasome subunit beta type-5	PSMB5	28.5	-	-	-	-	-	-
**Q9P0G3**	**Kallikrein-14**	**KLK14**	**29.1**	-	-	-	-	-	-
**P62258 ***	**14-3-3 protein epsilon**	**YWHAE**	**29.2**	-	x	-	-	x	x
P61247	Small ribosomal subunit protein eS1	RPS3A	29.9	-	-	-	-	-	-
P16152 *	Carbonyl reductase [NADPH] 1	CBR1	30.4	-	x	-	x	x	x
P21796 *	Voltage-dependent anion-selective channel protein 1	VDAC1	30.8	-	x	-	-	x	x
P27105 *	Stomatin	STOM	31.7	-	x	x	-	-	-
Q15181 *	Inorganic pyrophosphatase	PPA1	32.6	-	x	-	-	-	-
P52907 *	F-actin-capping protein subunit alpha-1	CAPZA1	32.9	-	-	-	-	x	-
**P06753 ***	**Tropomyosin alpha-3 chain**	**TPM3**	**32.9**	-	x	x	x	x	x
P09758	Tumor-associated calcium signal transducer 2	TACSTD2	35.7	-	-	-	-	-	-
Q13011	Delta(3,5)-Delta(2,4)-dienoyl-CoA isomerase, mitochondrial	ECH1	35.8	-	-	-	-	-	-
P13716 *	Delta-aminolevulinic acid dehydratase	ALAD	36.3	-	x	-	-	x	x
P14550	Aldo-keto reductase family 1 member A1	AKR1A1	36.5	-	-	-	-	-	-
P07195 *	L-lactate dehydrogenase B chain	LDHB	36.6	x	x	x	x	x	x
**Q15365 ***	**Poly(rC)-binding protein 1**	**PCBP1**	**37.5**	-	x	-	-	-	-
O14745 *	Na(+)/H(+) exchange regulatory cofactor NHE-RF1	CTSB	38.8	-	-	-	x	-	x
P15104 *	Glutamine synthetase	GLUL	42.0	x	x	x	x	x	x
**P08727**	**Keratin, type I cytoskeletal 19**	**KRT19**	**44.1**	-	-	-	-	-	-
P11279	Lysosome-associated membrane glycoprotein 1	LAMP1	44.9	-	-	-	-	-	-
P41218	Myeloid cell nuclear differentiation antigen	MNDA	45.8	-	-	-	-	-	-
O60235	Transmembrane protease serine 11D	TMPRSS11D	46.2	-	-	-	-	-	-
Q86T26	Transmembrane protease serine 11B	TMPRSS11B	46.3	-	-	-	-	-	-
Q9HDC9	Adipocyte plasma membrane-associated protein	APMAP	46.5	-	-	-	-	-	-
**O75874**	**Isocitrate dehydrogenase [NADP] cytoplasmic**	**IDH1**	**46.6**	-	-	-	-	-	-
P12532 *	Creatine kinase U-type, mitochondrial	CKMT1A	47.0	-	x	-	x	x	x
Q9UL52	Transmembrane protease serine 11E	TMPRSS11E	47.7	-	-	-	-	-	-
P23526 *	Adenosylhomocysteinase	AHCY	47.7	-	x	-	-	x	x
P22695 *	Cytochrome b-c1 complex subunit 2, mitochondrial	UQCRC2	48.4	-	x	-	-	-	x
**P13646**	**Keratin, type I cytoskeletal 13**	**KRT13**	**49.6**	-	-	-	-	-	-
Q9Y6N5	Sulfide:quinone oxidoreductase, mitochondrial	SQOR	49.9	-	-	-	-	-	-
Q9Y265	RuvB-like 1	RUVBL1	50.2	-	-	-	-	-	-
P55084	Trifunctional enzyme subunit beta, mitochondrial	HADHB	51.3	-	-	-	-	-	-
P49189 *	4-trimethylaminobutyraldehyde dehydrogenase	ALDH9A1	53.8	-	x	-	-	x	x
P00352 *	Aldehyde dehydrogenase 1A1	CAP1	54.8	-	x	-	-	x	x

**P43490** *	**Nicotinamide phosphoribosyltransferase**	**NAMPT**	**55.5**	-	x	-	x	x	x
**P19013**	**Keratin, type II cytoskeletal 4**	**KRT4**	**56.1**	-	-	-	-	-	-
P05091 *	Aldehyde dehydrogenase, mitochondrial	ALDH2	56.3	-	-	-	-	-	x
P21281 *	V-type proton ATPase subunit B, brain isoform	VATB2	56.5	-	x	-	x	x	x
Q8N1N4	Keratin, type II cytoskeletal 78	KRT78	56.8	-	-	-	-	-	-
P22307	Sterol carrier protein 2	SCP2	59.0	-	-	-	-	-	-
P25705 *	ATP synthase subunit alpha, mitochondrial	ATP5F1A	59.7	-	x	x	x	x	x
**P10809** *	**60 kDa heat shock protein, mitochondrial**	**HSPD1**	**61.0**	x	x	x	x	x	x
Q9UBL6	Copine-7	CPNE7	70.2	-	-	-	-	-	-
P13797	Plastin-3	PLS3	70.8	-	-	-	-	-	-
Q9UM07	Protein-arginine deiminase type-4	PADI4	74.0	-	-	-	-	-	-
**P02545** *	**Prelamin-A/C**	**LMNA**	**74.1**	-	-	-	x	-	x
O95171	Sciellin	SCEL	77.5	-	-	-	-	-	-
Q12931	Heat shock protein 75 kDa, mitochondrial	TRAP1	80.1	-	-	-	-	-	-
P07384	Calpain-1 catalytic subunit	CAPN1	81.8	-	-	-	-	-	-
**P08238** *	**Heat shock protein HSP 90-beta**	**HSP90AB1**	**83.2**	x	x	x	x	x	x
**P07900** *	**Heat shock protein HSP 90-alpha**	**HSP90AA1**	**84.6**	x	x	x	x	x	x
P05107	Integrin beta-2	ITGB2	84.7	-	-	-	-	-	-
P22735	Protein-glutamine gamma-glutamyltransferase K	TGM1	89.7	-	-	-	-	-	-
Q8WUM4	Programmed cell death 6-interacting protein	PDCD6IP	96.0	-	-	-	-	-	-
P06737	Glycogen phosphorylase, liver form	PYGL	97.1	-	-	-	-	-	-
O43707 *	Alpha-actinin-4	ACTN4	104.8	-	x	-	-	x	-
**P22314** *	**Ubiquitin-like modifier-activating enzyme 1**	**UBA1**	**117.8**	-	x	-	-	x	x
**P11215**	**Integrin alpha-M**	**ITGAM**	**127.1**	-	-	-	-	-	-
P46940	Ras GTPase-activating-like protein IQGAP1	IQGAP1	189.1	-	-	-	-	-	-
O60437	Periplakin	PPL	204.6	-	-	-	-	-	-
**P35579** *	**Myosin-9**	**MYH9**	**226.4**	-	x	x	x	x	x

*Proteins previously identified in GBM CUSA fluid [22].
#Cancer related classified proteins are marked in bold.
§Proteins classified as candidate biomarkers in The Human Protein Atlas database are underlined.
