# Supplementary material for: Proteomic Profiling of Pre- and Post-Surgery Saliva of Glioblastoma Patients: A Pilot Investigation
Source: Int J Mol Sci. 2024 Dec 3;25(23):12984. doi: 10.3390/ijms252312984 (PMC11641718; doi:10.3390/ijms252312984)
Supplement: Supplementary file 1 [file ijms-25-12984-s001.zip › Table S3.pdf]

Table S3. List of the 22 proteins exclusively identified in R_T0 saliva. Proteins are listed in order of increasing molecular mass (kDa). Proteins identification data in CUSA fluid from different zones, namely tumor CORE and 5-aminolevulinic induced fluorescence positive (A+) and negative (A-) peripheral zones, are also included [22].

Accession*	Description #§£	Gene name	kDa	CUSA ND CORE	CUSA ND A+	CUSA ND A-	CUSA R CORE	CUSA R A+	CUSA R A-
P22532	Small proline-rich protein 2D	SPRR2D	7.9	-	-	-	-	-	-
Q9BPY8	Homeodomain-only protein	HOPX	8.3	-	-	-	-	-	-
**P29034**	**Protein S100-A2**	**S100A2**	**11.1**	-	-	-	-	-	-
Q9UJC5	SH3 domain-binding glutamic acid-rich-like protein 2	SH3BGRL2	12.3	-	-	-	-	-	-
P01624*	Immunoglobulin kappa variable 3-15	IGKV3	12.5	-	-	-	-	x	-
O60361	Putative nucleoside diphosphate kinase	NME2P1	15.5	-	-	-	-	-	-
P39019	Small ribosomal subunit protein eS19	RPS19	16.1	-	-	-	-	-	-
Q13404	Ubiquitin-conjugating enzyme E2 variant 1	UBE2V1	16.5	-	-	-	-	-	-
Q969H8	Myeloid-derived growth factor	MYDGF	18.8	-	-	-	-	-	-
P55145	Mesencephalic astrocyte-derived neurotrophic factor	MANF	20.7	-	-	-	-	-	-
**P55327**	**Tumor protein D52**	**TPD52**	**24.3**	-	-	-	-	-	-
P51858	Hepatoma-derived growth factor	HDGF	26.8	-	-	-	x	-	-
Q9NQR4	Omega-amidase NIT2	NIT2	30.6	-	-	-	-	-	-
P22676	Calretinin	CALB2	31.5	-	-	-	-	-	-
Q9BQR3	Serine protease 27	PRSS27	31.9	-	-	-	-	-	-
Q01459	Di-N-acetylchitobiase	CTBS	43.7	-	-	-	-	-	-
**P17174***	**Aspartate aminotransferase, cytoplasmic**	**GOT1**	46.2	-	x	x	-	x	x
Q13231	Chitotriosidase-1	CHIT1	51.6	-	-	-	-	-	-
P10619	Lysosomal protective protein	CTSA	54.4	-	-	-	-	-	-
O75131	Copine-3	CPNE3	60.1	-	-	-	-	-	-
P0CG48*	Polyubiquitin-C	UBC	77.0	-	x	-	-	x	x
O75882	Attractin	ATRN	158.4	-	-	-	-	-	-

*Proteins previously identified in GBM CUSA fluid [22].
#Cancer related classified proteins are marked in bold.
§Proteins classified as candidate cancer biomarkers in The Human Protein Atlas database are underlined.
